# Formation of pyramidal structures through mixing gold and platinum atoms: the Au_*x*_Pt_*y*_^2+^ clusters with *x* + *y* = 10†

**DOI:** 10.1039/d3ra06000d

**Published:** 2023-11-08

**Authors:** Bao-Ngan Nguyen-Ha, Cam-Tu Phan Dang, Long Van Duong, My Phuong Pham-Ho, Minh Tho Nguyen, Nguyen Minh Tam

**Affiliations:** a Laboratory for Chemical Computation and Modeling, Institute for Computational Science and Artificial Intelligence, Van Lang University Ho Chi Minh City Vietnam ngan.nguyenhabao@vlu.edu.vn minhtho.nguyen@vlu.edu.vn; b Faculty of Applied Technology, School of Technology, Van Lang University Ho Chi Minh City Vietnam; c Faculty of Natural Sciences, Duy Tan University Da Nang Vietnam; d Institute of Research and Development, Duy Tan University Da Nang Vietnam; e Atomic Molecular and Optical Physics Research Group, Science and Technology Advanced Institute, Van Lang University Ho Chi Minh City Vietnam; f Faculty of Chemical Engineering, Ho Chi Minh City University of Technology (HCMUT) 268 Ly Thuong Kiet Street, District 10 Ho Chi Minh City Vietnam; g Vietnam National University Ho Chi Minh City Linh Trung Ward, Thu Duc City Ho Chi Minh City Vietnam; h Faculty of Basic Sciences, University of Phan Thiet 225 Nguyen Thong Phan Thiet City Binh Thuan Vietnam nmtam@upt.edu.vn

## Abstract

The geometric and electronic structures of a small series of mixed gold and platinum Au_*x*_Pt_*y*_^2+^ clusters, with *x* + *y* = 10, were investigated using quantum chemical methods. A consistent tetrahedral pyramid structure emerges, displaying two patterns of structural growth by a notable critical point at *y* = 5. This affects the clusters' electron population, chemical bonding, and stability. For the Pt-doped Au clusters with *y* values from 2 to 5, the bonds enable Pt atoms to assemble into symmetric line, triangle, quadrangle, and tetragonal pyramidal Pt_*y*_ blocks, respectively. For the Au-doped Pt clusters, with larger values of *y* > 5, the structures are more relaxed and the d electrons of Pt atoms become delocalized over more centers, leading to lower symmetry structures. A certain aromaticity arising from delocalization of d electrons over the multi-center framework in the doped Pt clusters contributes to their stability, with Pt_10_^2+^ at *y* = 10 exhibiting the highest stability. While the ground electronic state of the neutral platinum atom [Xe]. 4f^14^5d^9^6s^1^ leads to a triplet state (^3^D_3_), the total magnetic moments of Au_*x*_Pt_*y*_^2+^ are large increasing steadily from 0 to 10 *μB* and primarily located on Pt atoms, corresponding to the increase of the number of Pt atoms from 0 to 10 and significantly enhancing the magnetic moments. An admixture of both Au and Pt atoms thus emerges as an elegant way of keeping a small pyramidal structure but bringing in a high and controllable magnetic moment.

## Abstract 2

The geometric and electronic structures of a small series of mixed gold and platinum Au_*x*_Pt_*y*_^2+^ clusters, with *x* + *y* = 10, were investigated using quantum chemical methods.

## Graphical abstract

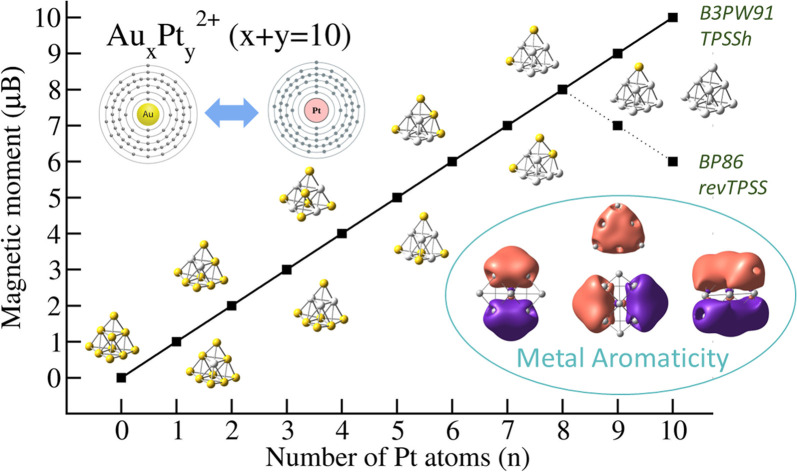

## Introduction

The element gold and gold-based clusters have several unique electronic,^1–3^ optical,^4–8^ chemical,^9,10^ and catalytic^11–15^ properties that have been, and still are, triggering an explosive growth in both experimental and theoretical studies so far. Gold has an electronic configuration of 5d^10^6s^1^ but in contrast to other coinage metal atoms, the gold atom exhibits a typical sd hybridization, resulting from a strong relativistic effect of the heavy element^16^ and emphasizing numerous specific and customized properties, particularly in its geometric and electronic structures. As a result, stable frameworks of pure gold clusters with intriguing structures have emerged such as 2D-planar,^17^ flat cage,^18,19^ tubular,^20^ icosahedral,^21–24^ core–shell,^25^ star-like shape,^26,27^ and tetrahedral structures. In the latter, the tetrahedron stands out by a completely filled electronic shell.^16,28–30^

Through the use of a combination of photoelectron spectroscopic techniques and relativistic density functional theory (DFT) calculations, a tetrahedral cluster of 20 gold atoms was identified as an ideal building block for gold surfaces.^31^ The distinctive Au_20_ cluster exhibits a notably large HOMO–LUMO energy gap (1.8 eV) exceeding that of the well-known C_60_ fullerene. This characteristic contributes to the cluster's exceptional inertness and stability in both its geometric and electronic structures. The Au_20_ pyramid can also be considered as a superatom with an electronic shell-closure. Its distinctive features include a (*16c-16e*) superatomic Au-core connected to four vertical Au atoms *via* a SD^3^ hybridization,^16^ resulting in a closed electron shell configuration of (1S^2^1P^6^2S^2^1D^10^) of Au_20_ giving rise to its magic number of 20 valence electrons.^28^ This pyramidal superatomic cluster could be used as building blocks for assembling cluster-based materials.

**Scheme 1:**
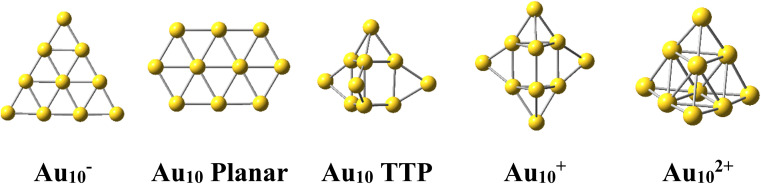
Shapes of the Au_10_ cluster in the anionic, neutral, cationic and dicationic states.

The Au_10_ clusters in different charge states were expected to also have a tetrahedral skeleton like Au_20_. However, it was found that the neutral Au_10_ favors a 2D planar structure of elongated hexagon^17^ (Scheme 1). Recently, Nhat *et al.*^32^ reported that both the 2D elongated hexagonal and 3D tetra-capped trigonal prism (TTP) isomers of Au_10_ are likely to exist together in experimental molecular beams at temperatures between 100 and 300 K.^32^ On the other hand, the Au_10_^−^ monoanion was observed to prefer a 2D-planar structure,^1,33,34^ whereas the Au_10_^+^ monocation possesses a quasi-tetrahedral TTP shape^35–37^ (Scheme 1). It is well known that while the negative charge tends to favour the planar form of atomic clusters, the positive charge induces 3D shapes.

For its part, the dicationic Au_10_^2+^ cluster is of particular interest due to its magic number of 8 valence electrons, which forms a closed electron shell of (1S^2^1P^6^) and an exceptionally large HOMO–LUMO energy gap of 3.9 eV.^29^ The geometrical structure of Au_10_^2+^ dication has theoretically been determined to be a tetrahedral pyramid with *T*_d_ symmetry^29^ (Scheme 1) and is considered as a tetravalent SP^3^-Au_6_ core decorated with four capping Au atoms.^38^ The coincidence of both the same geometric symmetry and a magic electron shell makes both Au_10_^2+^ and Au_20_ clusters equivalent superatoms.^30^ However, this magic character invariably causes these clusters to have no magnetic properties.

In order to induce magnetism in these clusters, the possibility of incorporating additional elements, particularly transition metals, into the gold tetrahedral Au_20_ and Au_10_^2+^ clusters was considered.^30,39,40^ While doping of some transition metals can cause significant geometric transformations to form endohedral structures, the M@Au_19_ having 19-Au atoms doped with lighter transition metals such as Cr, Mn, Fe, Co, Ni and Cu still maintain their tetrahedral framework.^39^ Among these clusters, CrAu_19_ exhibits the largest magnetic moment of 5 *μ*_B_ with its 20 delocalized valence electrons forming a stable electron shell of (1S^2^1P^6^2S^2^1D^10^) and the 3d-Cr atomic shell is partially filled by its five remaining localized electrons, resulting in a total magnetic moment of 5 *μ*_B_.^40^ A recent investigation^29^ explored the effects of first-row transition metal M doping on the Au_9_^2+^ dication and found that the total spin magnetic moment of the metal doped MAu_9_^2+^ dication is induced mainly by electrons on the 3d-AOs of dopant M atoms and is, as expected, strongly dopant-dependent, varying from the smallest value of 0 *μ*_B_ for ScAu_9_^2+^ to the largest value of 5 *μ*_B_ for CrAu_9_^2+^.^30^ Despite shape variations in ScAu_9_^2+^ and TiAu_9_^2+^, the tetrahedral framework is maintained in the remaining MAu_9_^2+^ clusters.

The platinum element which has an electronic configuration of [Xe]4f^14^5d^9^6s^1^ and a triplet ground state (^3^D_3_), is located next to gold on the Periodic Table. Unlike the gold Au_10_, the pure platinum Pt_10_ cluster exhibits a highly stable tetrahedral shape and is regarded as a magic cluster^41^ at a nonet spin state.^42,43^ A planar isomer of Pt_10_ is much higher in energy. For its part, the Pt_10_^−^ anion has been reported to follow the [Pt_6_@Pt_4_]^−^ model,^44^ which, similar to Au_10_^2+^, imitates the tetrahedral arrangement of the methane molecule with the Pt_6_ core substituting for the carbon center. What makes this even more intriguing is that both the significant magnetic moment and tetrahedral pyramid framework are present in all three charged states of Pt_10_.^45–47^ This suggests that Pt could be a suitable dopant which could either preserve the tetrahedral framework and/or improve the magnetic moment of gold cluster through its unpaired electrons. In other words, substitution of Au atom(s) in the pure Au_10_^2+^ dication by Pt atom(s) in a stepwise manner, could open the Au_10_^2+^ closed electron shell and thereby increase its magnetic moment while keeping the pyramidal shape. In this context, we set out to perform a detailed and systematic investigation on the binary Au_*x*_Pt_*y*_^2+^ clusters with *x* + *y* = 10, making use of quantum chemical computations to scrutinize their geometries as well as their corresponding electronic and magnetic properties. It is clear that when *x* < *y*, the Au atom plays the role of dopant on Pt clusters. Our goal is to understand the factors governing the formation of a tetrahedral pyramid as the most stable isomer following mixture of a small number of Au and Pt atoms.

### Computational methods

All standard electronic structure calculations are performed using the Gaussian 09 package.^48^ In theoretical studies of the heavy atoms and their cluster systems, it is important to properly account for relativistic effects,^15,23,37,49–51^ in particular that of the gold atom.^15,23,52^ This is in fact an issue of concern, because in density functional theory (DFT) methods, even they are favored over wavefunction methods thanks to their lower computational cost and decently accurate outcome, there is no global functional which can accurately describe the properties of both Au and Pt atoms.^37,53^ Several DFT functionals have been applied to these metals including the BP86,^33,35^ TPSS,^54^ BPE,^55,56^ BLYP,^32^ B2PLYP,^37^ M0*x* (*x* = 5, 6, 8) as well as M0*x* with variously modified Hartree–Fock exchange^57^ such as M05-2X,^58^ M06-L,^59^*etc.* to interpret the experimental results of transition metals.^19,33,35,37,54–57^

In previous studies of dicationic gold clusters^29^ as well as both pure and doped Pt clusters,^42,60–63^ the hybrid B3PW91 functional has been extensively used. This functional has been well calibrated and has demonstrated good agreement with experimental results for doped Pt clusters.^61^ Furthermore, the BP86 and revTPSS functionals are also commonly employed when studying Au and transition metal (TM)-doped Au clusters,^32,64^ while the TPSSh functional has been utilized for Pt_n_ clusters.^43,65^ Hence to ensure consistency and facilitate comparison, we employ the B3PW91, BP86, TPSSh, and revTPSS functionals in our current investigation to explore stable species of the binary clusters Au_*x*_Pt_*y*_^2+^ with *x* + *y* = 10.

The low-lying isomers of a cluster are located on the corresponding potential energy surface which is explored using an intensive search procedure covering as many atomic arrangements as possible. Our search for energy minima of clusters is conducted using two different approaches. First, all possible structures of Au_*x*_Pt_*y*_^2+^ clusters are generated using a stochastic algorithm^66^ which was improved from the previously reported random kick procedure.^67^ By another way, initial structures of Au_*x*_Pt_*y*_^2+^ are manually built by either replacing *y* Pt atoms into various positions belonging to local minimum structures of both neutral and charged states of Au_10_,^1,17,29,32–35^ or doping *y* Pt atoms onto the surfaces of the pure Au_*x*_^+/0/−^ clusters,^1,2,17,33–35^ and conversely by doping *x* Au atoms into the Pt_*y*_^+/0/−^ clusters alike.^42,60^ These initial structures are first optimized at different multiplicities using the B3PW91, BP86, TPSSh, and revTPSS functional in conjunction with the small LANL2DZ basis set. Subsequently, the local energy minima identified from both search approaches with relative energies of <5 eV with respect to the lowest-lying minimum of each size are all re-optimized using the same functional but with the larger aug-cc-pVTZ-PP basis set, in which PP stands for an effective core potential including the relativistic effects for heavy atoms. Harmonic vibrational frequencies are then calculated at the same level to identify equilibrium geometries and evaluate their zero-point energy (ZPE) corrections.

The natural bond orbital (NBO) analysis is performed by the NBO 5.0 program^68^ to investigate the electronic configurations and populations thereby the chemical bonding and magnetic properties of the clusters considered. The results of NBO calculation are further analyzed^69^ using the Multiwfn program^70^ to study bonding characteristics of the Au_*x*_Pt_*y*_^2+^ clusters.

## Results and discussion

### Lower-lying structures

**Fig. 1:**
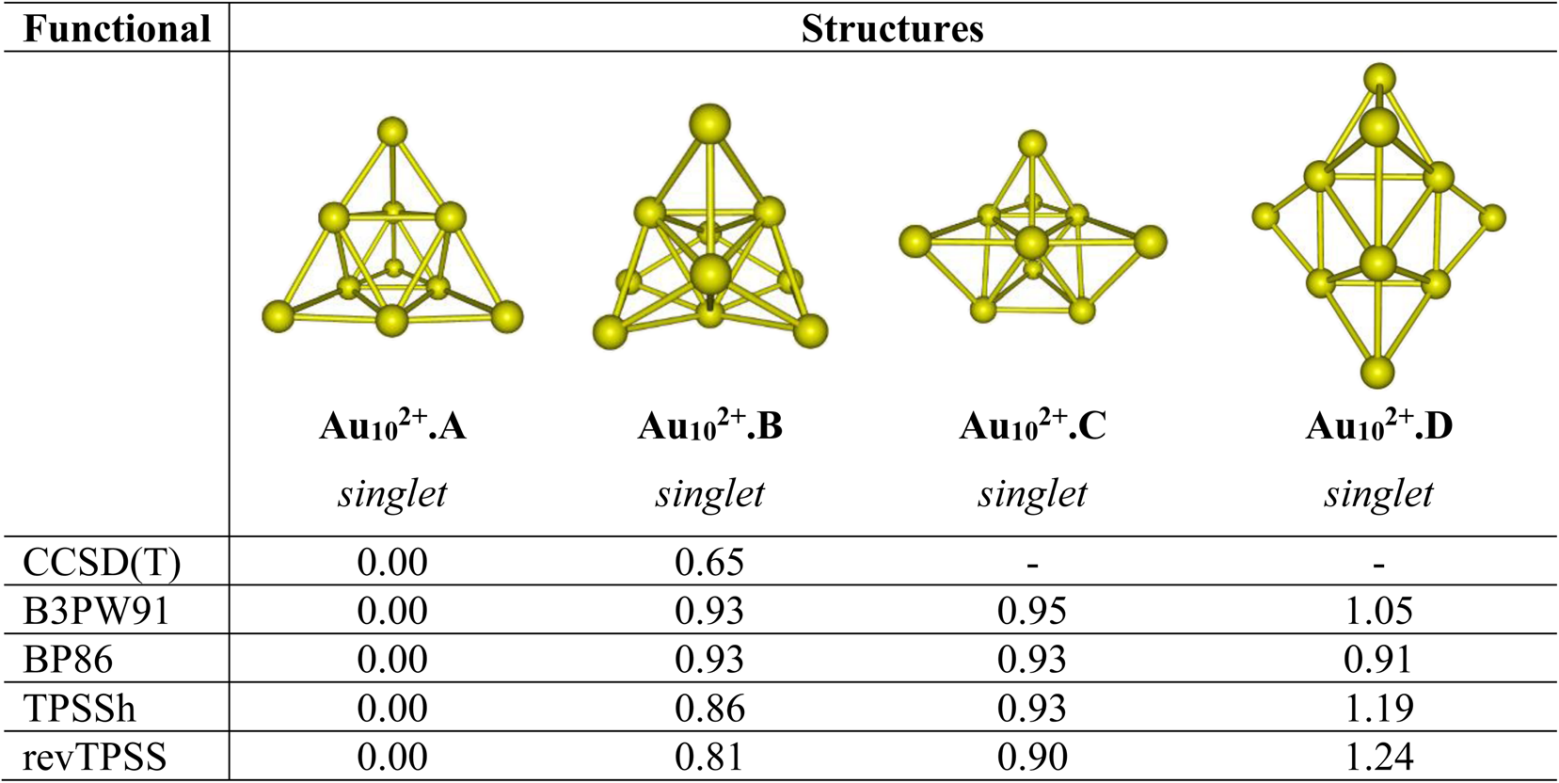
Relative energies Δ*E* (eV) of the low-lying Au_10_^2+^ isomers using CCSD(T)/cc-pVDZ-PP and B3PW91, TPSSh, BP86 and revTPSS functionals with the aug-cc-pVTZ-PP bais set and ZPE corrections.

Let us first evaluate the performance of difference density functionals on the relative energies between isomers. Fig. 1 illustrates the assessment of low-lying structures within the Au_10_^2+^ cluster, by combining the CCSD(T) method with the cc-pVDZ-PP basis set and the B3PW91, BP86, TPSSh, and revTPSS functionals in conjunction with the aug-cc-pVTZ-PP basis set. The results unequivocally support the conclusion that the pyramidal configuration stands out as the sole and most stable structure for the Au_10_^2+^ clusters.

**Fig. 2:**
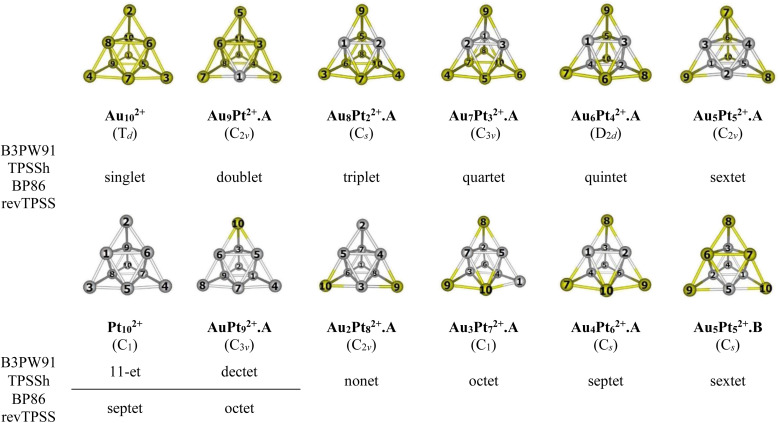
Structures, geometries (in bracket) and spin states of the lowest-lying Au_*x*_Pt_*y*_^2+^ (*x* + *y* = 10) at each size using B3PW91, TPSSh, BP86 and revTPSS/aug-cc-pVTZ-PP + ZPE computations. Yellow ball = Au and gray ball = Pt.

For the Au_*x*_Pt_*y*_^2+^ (*x* + *y* = 10), due to the abundance of local minima on the potential energy surface for each cluster size, we selectively include only the lowest-lying isomers with relative energies in proximity to the most stable structure (with a difference of <1.00 eV in relative energy).^71,72^ The shapes of the Au_*x*_Pt_*y*_^2+^ equilibrium structures, their spin states and their relative energies obtained at the B3PW91, BP86, TPSSh, or revTPSS with the aug-cc-pVTZ-PP basis set and ZPE corrections are displayed in Fig. 2, S1 and S2 (ESI file).†

As for a convention, the Au_*x*_Pt_*y*_^2+^.Z label is used to denote the isomers where *x* is the number of Au atoms, *y* the number of Pt atoms and *Z* = *A*, *B*, *C*,… accords to the isomers with increasing relative energy. Hence, the notation Au*_x_*Pt*_y_*^2+^.A invariably stands for the most stable isomer A of the Au_*x*_Pt_*y*_^2+^ dication.

In general, regardless of the specific numbers of Au or Pt atoms (represented by *x* and *y*) and the calculation methods employed, it has been observed that the most stable isomer for each size (*x*, *y*) consistently exhibits a tetrahedral pyramid structure (Fig. 2). This indicates a strong preference for this geometric skeleton within this series of small binary clusters. Previous studies suggested that Au_10_^2+^ comprises a hybridized tetravalent SP^3^-Au_6_^2+^ core with an octahedral central structure, adorned by four extra Au atoms on the Au_3_ faces of this core (Scheme 1).^29,38^ However, in the case of Pt-doped Au clusters, the use of a [Au_6_X_4_]^2+^ model^38^ cannot adequately explain the shapes of these Au_*x*_Pt_*y*_^2+^ dications. This is due to the fact that Au atoms in core positions are successively replaced by Pt atoms, even before the Au atoms at the top positions of the tetrahedron (*cf.*Fig. 2). This is because (i) the Pt–Pt bond is stronger than the Au–Au bond (*cf.* section “thermodynamic stability” hereafter) and (ii) d-electrons of Au atoms do not join to a formation of multi-center bonds, but d-electrons of Pt atoms do (*cf.* section “chemical bonding” hereafter). In other words, Pt atoms tend to gather into small groups in such a way that their electrons can move more freely around them. Such a preference for Pt atoms placed in the core and Au atoms at the surface can also explain why the core Au_6_ is not fixed in place.

Accordingly, two tendencies emerge in the formation of binary structures with a critical point at size *x* = *y* = 5. In the first trend, the Pt_*y*_ blocks in the lowest-lying structures Au_9_Pt^2+^.A, Au_8_Pt_2_^2+^.A, Au_7_Pt_3_^2+^.A, Au_6_Pt_4_^2+^.A and Au_5_Pt_5_^2+^.A regularly develop from a spot, a line, a triangle, a quadrangle and a tetragonal pyramid as *y* increases from 1 to 5, respectively (*cf.*Fig. 2). Each of these blocks constitutes the octahedral center which is located inside the pyramid. However, in the second trend of *y* = 6–9, replacement of Pt no longer follows the same pattern of structural growth as that of *y* = 1–5, and some Pt atoms tend to occupy outer vertices rather than apexes of the inner core (*cf.*Fig. 2). In fact, in this series where *y* > *x*, the mixed systems can better be regarded as Au-doped Pt clusters, and Pt atoms assemble forming the cores of the pyramid. The lowest-lying structures at sizes *y* = 6–9, namely Au_4_Pt_6_^2+^.A, Au_3_Pt_7_^2+^.A, Au_2_Pt_8_^2+^.A and AuPt_9_^2+^.A follow a similar pattern that can be traced back to the shape of the Au_5_Pt_5_^2+^.B isomer and are achieved by sequentially replacing the remaining Au atoms in Au_5_Pt_5_^2+^.B by Pt atoms. It is worth noting that both isomers Au_5_Pt_5_^2+^.A and Au_5_Pt_5_^2+^.B are in competition for the global minimum structure, given their small relative energy gap of only ∼0.1eV with a marginal energy preference for the Au_5_Pt_5_^2+^.A (*cf.* Fig. S2†). This energy gap is, in fact, smaller than the expected error margin of ±0.3 eV, typically associated with energetic parameters derived from DFT computations.^71,72^ To further validate these findings, the energies of these two isomers are also calculated using the CCSD(T)/cc-pVDZ-PP method, yielding results consistent with an energy difference of ∼0.1 eV. This additional analysis reinforces the assumption of energy degeneracy between the two geometric structures.

In the transitional size, five Au atoms of Au_5_Pt_5_^2+^.B are arranged as a triangle corner, where two atoms reside at the core, while the remaining three atoms occupy the outer vertices. This triangular arrangement forms one of the three faces of a tetrahedron (*cf.*Fig. 2). Following the same framework as Au_5_Pt_5_^2+^.B, the Au_4_Pt_6_^2+^.A isomer which has more Pt atom than Au, is formed by replacing one of the two Au atoms at the core of the Au_5_Pt_5_^2+^.B with a Pt atom. Consequently, Au_4_Pt_6_^2+^.A is now composed of five Pt atoms in the internal core and one Pt atom locating at an external vertex and with Au atoms serving as dopants (*cf.*Fig. 2). This substitution leads to a relaxation towards *C*_s_ symmetry.

A similar phenomenon is witnessed in the Au_3_Pt_7_^2+^.A, where the substitution of Au atoms with Pt atoms takes place at an external vertex of Au_4_Pt_6_^2+^.A, resulting in the attainment of *C*_1_ symmetry. Continuing this trend, replacing the last Au atom at the internal core of the Au_3_Pt_7_^2+^.A with a Pt atom, we obtain the Au_2_Pt_8_^2+^.A. By further sequentially substituting Au atom(s) in Au_2_Pt_8_^2+^.A with Pt atom(s), we eventually arrive at the AuPt_9_^2+^.A and the Pt_10_^2+^ clusters (*cf.*Fig. 2).

### Thermodynamic stability

The B3PW91/aug-cc-pVTZ-PP + ZPE method is utilized to investigate the thermodynamic stability of the clusters. The inherent thermodynamic stability of the 10-atom clusters is evaluated by the average binding energies (*E*_b_). In this work, the average binding energies (*E*_b_) of all lowest-lying structures of Au_*x*_Pt_*y*_^2+^ can conventionally be defined using the following formula (eqn (1)–(3)):

1 *E*_b_(Au_*x*_Pt_*y*_^2+^) = [*xE*(Au) + (*y* − 2)*E*(Pt) + 2*E*(Pt^+^) − *E*(Au_*x*_Pt_*y*_^2+^)]/10

Particularly for two dicationic clusters Au_10_^2+^ and AuPt_9_^2+^, the *E*_b_ can be defined as eqn (2) and (3), respectively:

2 *E*_b_(Au_10_^2+^) = [8*E*(Au) + 2*E*(Au^+^) − *E*(Au_10_^2+^)]/10

**Fig. 3:**
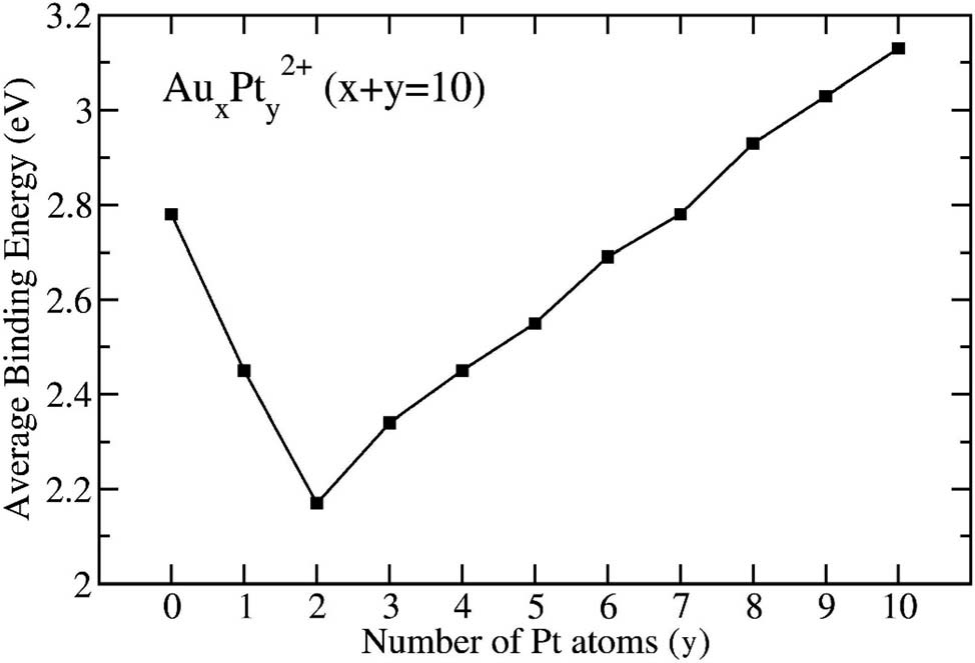
Average binding energies of Au_*x*_Pt_*y*_^2+^ clusters with *x* + *y* = 10, obtained at the B3PW91/aug-cc-pVTZ-PP + ZPE level.

3 *E*_b_(Au_9_Pt^2+^) = [8*E*(Au) + *E*(Au^+^) + *E*(Pt^+^) − *E*(Au_9_Pt^2+^)]/10

where *E*(Au), *E*(Au^+^), *E*(Pt), *E*(Pt^+^), and *E*(Au_*x*_Pt_*y*_^2+^) represent the total energies of the Au-atom, the cationic Au^+^, the Pt atom, the cationic Pt^+^, and the dicationic Au_*x*_Pt_*y*_^2+^, respectively. Given that the ionization energy of the Au atom (13.03 eV) significantly exceeds that of the Pt atom (9.18 eV), we opt to utilize only the total energies of the Pt^+^ cation *E*(Pt^+^) for calculating the average binding energy instead of the total energies of the Au^+^ cation *E*(Au^+^). This replacement is grounded in the assumption that the loss of two electrons takes place on Pt atoms, rather than one Au atom and one Pt atom, elucidating the inclusion of the 2*E*(Pt^+^) term in formula (1). The plots of *E*_b_ illustrating their evolution are depicted in Fig. 3.

The mixed clusters exhibit varying levels of stability, with the Au_8_Pt_2_^2+^ cluster being the least stable due to energetic degeneracy between singlet and triplet states. Mixed clusters with a limited number of Pt atoms (*y* = 1–6) exhibit lower stability as compared to the original Au_10_^2+^. This could be attributed to the insufficiency of Pt–Pt bonds due to the limited number of Pt atoms and the deficiency of multi-center bonds formed between atomic orbitals (AOs) of Pt and Au atoms. In these cases, some distortion in the overall bonds *(10c-1e)* occurs because of the contamination in S and P-MOs within the mixed clusters in comparison to the pure Au_10_^2+^ clusters (*cf.* section “chemical bonding” hereafter).

However, as the number of Pt atoms increases beyond *y* = 2, the mixed clusters exhibit a linear stabilization (see Fig. 3). Notably, starting at *y* = 7, where it resembles a doped Pt cluster, the stability of the mixed clusters surpasses that of the Au_10_^2+^ cluster. At *y* = 10, which corresponds to a pure Pt cluster, Pt_10_^2+^ emerges as the most stable configuration. This suggests that a Pt cluster, even with the same number of atoms as its Au counterpart, is inherently more stable.

The underlying reason for this enhanced stability lies in the intrinsic strength of the Pt–Pt bond, boasting a bond energy of 2.91 eV, as compared to the Au–Au (2.03 eV) and Au–Pt (2.04 eV) bond energy. As the number of Pt atoms increases, this strength is amplified, resulting in the formation of more robust Pt–Pt bonds. Consequently, these stronger bonds contribute to greater stability within the mixed clusters.

It's worth noting that the prevalence of stronger Pt–Pt bonds leads Pt atoms to congregate together, forming small Pt blocks within the clusters. This arrangement facilitates the movement of d-electrons between the Pt atoms, resulting in a creation of smaller aromatic regions characterized by free d-electrons in the Pt blocks. These exists alongside the primary aromaticity arising from in S and P-MOs throughout the entire Au_*x*_Pt_*y*_^2+^ clusters (*cf.* section “chemical bonding” hereafter).

### Magnetic moments

In terms of spin state, the total and local magnetic moments (TMMs and LMMs, respectively) are determined by calculating the difference between the number of spin-up and spin-down electrons occupying the molecular or atomic orbitals.

**Fig. 4:**
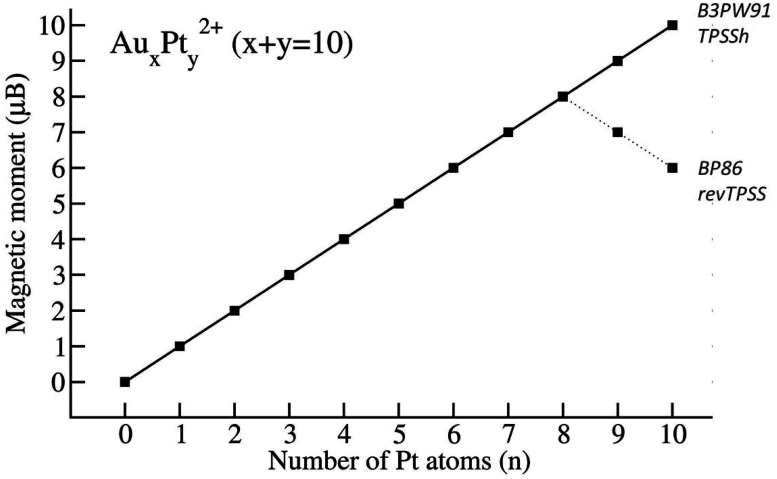
Magnetic moments of Au_*x*_Pt_*y*_^2+^ clusters with *x* + *y* = 10.

When using the B3PW91 and TPSSh functionals, an increase in the number of Pt atoms in the mixed clusters, due to the intrinsic triplet ground state of Pt atom, invariably leads to a higher multiplicity of their most stable isomers. In other words, as the size of Pt increases from 0 to 10, the corresponding total magnetic moment of the Au_*x*_Pt_*y*_^2+^ steadily increases from 0 to 10 *μ*_B_ (*cf.*Fig. 4). The electronic configuration of pure Au_10_^2+^, as mentioned earlier, is a closed-shell singlet state with four bonds formed by the SP^3^-Au_6_^2+^ core bonded with four 6s electrons of four external Au atoms using the eight shared valence electrons,^38^ leading to a completely quenched total magnetic moment. When Pt atoms replace Au in Au_10_^2+^, the closed-shell electronic configuration becomes defective due to the deficiency of one electron on each Pt 5d-AO. NBO results confirm that the magnetic moment mainly localizes on Pt atoms (as shown in Table 1) with a spin density range of 0.7–1.2 electron for each Pt atom and the unpaired d-electron is fixed on each Pt atom. As each Pt atom donates one single electron, an increase in the number of Pt atoms results in an increasing number of unpaired electrons in the cluster, and thereby leads to a significant increase in magnetic moments.

Local and total spin magnetic moment of the binary Au_*x*_Pt_*y*_^2+^ (*x* + *y* = 10) obtained at B3PW91/aug-cc-pVTZ-PP theory level

**Table:** 

No	Au_9_Pt^2+^.A (doublet)		Au_8_Pt_2_^2+^.A (triplet)		Au_7_Pt_3_^2+^.A (quartet)		Au_6_Pt_4_^2+^.A (quintet)		Au_5_Pt_5_^2+^.A (sextet)	
1	Pt	0.8	Pt	1.0	Pt	0.9	Pt	0.9	Pt	0.9
2	Au	0.1	Pt	0.9	Pt	0.9	Pt	0.9	Pt	1.0
3	Au	0.0	Au	0.0	Pt	0.9	Pt	0.9	Pt	0.9
4	Au	0.0	Au	0.0	Au	0.1	Pt	0.9	Pt	0.9
5	Au	0.0	Au	0.1	Au	0.0	Au	0.0	Pt	0.9
6	Au	0.0	Au	0.0	Au	0.0	Au	0.0	Au	0.0
7	Au	0.1	Au	0.0	Au	0.0	Au	0.1	Au	0.1
8	Au	0.0	Au	0.0	Au	0.1	Au	0.1	Au	0.1
9	Au	0.0	Au	0.0	Au	0.1	Au	0.1	Au	0.1
10	Au	0.0	Au	0.0	Au	0.0	Au	0.1	Au	0.1
	Total	1.0	Total	2.0	Total	3.0	Total	4.0	Total	5.0

**Table:** 

No	Au_4_Pt_6_^2+^.A (septet)		Au_3_Pt_7_^2+^.A (octet)		Au_2_Pt_8_^2+^.A (nonet)		AuPt_9_^2^.A (dectet)		AuPt_9_^2+^.A (octet)	
1	Pt	1.0	Pt	0.8	Pt	0.7	Pt	1.2	Pt	1.0
2	Pt	1.0	Pt	1.0	Pt	0.7	Pt	0.7	Pt	0.3
3	Pt	1.1	Pt	1.0	Pt	1.0	Pt	1.1	Pt	1.1
4	Pt	1.0	Pt	1.2	Pt	1.1	Pt	0.7	Pt	0.4
5	Pt	0.7	Pt	1.1	Pt	1.1	Pt	1.1	Pt	1.1
6	Pt	1.0	Pt	0.7	Pt	1.1	Pt	1.1	Pt	1.1
7	Au	0.1	Pt	1.0	Pt	1.2	Pt	1.2	Pt	0.7
8	Au	0.1	Au	0.1	Pt	1.1	Pt	0.7	Pt	0.4
9	Au	0.1	Au	0.1	Au	0.1	Pt	1.2	Pt	1.0
10	Au	0.0	Au	0.1	Au	0.1	Au	0.0	Au	0.0
	Total	6.0	Total	7.0	Total	8.0	Total	9.0	Total	7.0

**Table:** 

No	Pt_10_^2+^ (11-et)		Pt_10_^2+^ (septet)	
1	Pt	1.2	Pt	1.1
2	Pt	0.7	Pt	0.5
3	Pt	0.7	Pt	0.5
4	Pt	0.7	Pt	0.5
5	Pt	1.2	Pt	1.1
6	Pt	1.2	Pt	1.1
7	Pt	1.2	Pt	0.5
8	Pt	1.2	Pt	0.5
9	Pt	1.2	Pt	0.5
10	Pt	0.7	Pt	−0.3
	Total	10.0	Total	6.0

However, when utilizing the BP86 and revTPSS methods, the magnetic moments reach their peak at *y* = 8 (eight Pt atoms) with a value of 8 *μB*, and then gradually decrease to 6 *μB* when *y* = 10 (ten Pt atoms) (*cf.*Fig. 4).

Both the Pt_10_^2+^ clusters with septet and 11-et spin states and the AuPt_9_^2+^ clusters with octet and dectet spin states, the relative energy gaps among all four DFT methods considered, as listed in Fig. S2,† are very small (<0.1 eV). This indicates that these spin states can be considered to be energetically degenerate, arising from the closely spaced energy levels of the d-orbitals within these clusters.

The high spin states exhibited by these clusters pose a challenge for the coupled-cluster method calculations as well, primarily due to an increasing spin contamination, resulting from its unrestricted Hartree–Fock (UHF) wavefunctions, whose spin contamination tends to lead to a slow and deceptive convergence of the coupled-cluster expansions, and thereby incorrect total energies. Consequently, determination of a reliable method for these systems becomes a complex task, in particular for system having multi-reference character.

### Chemical bonding

For a rationalization of the chemical bonding of these Au-Pt mixed clusters, the electronic configuration of each lowest-lying isomer Au*_x_*Pt*_y_*^2+^.A displayed in Fig. 2 is analyzed using NBO at the B3PW91/cc-pVTZ-PP theory level and related adaptive natural density partitioning (AdNDP) analysis.^70^ Conventionally, the (*nc-me*) label specifies the number *n* of atomic centers and the number *m* of electrons moving within those centers. For instance, (*10c-1e*) indicates one electron moving over ten atomic centers in the cluster.

The binary Au_*x*_Pt_*y*_^2+^ clusters, with *y* varying from 0 to 10, prominently exhibit a spherical aromaticity. This aromaticity arises from the presence of *(10c-2e)* bonds in case of the Au_10_^2+^ and *(10c-1e)* bonds in both the alpha and beta electron sides of the Au_*x*_Pt_*y*_^2+^ clusters (*y* = 1–10). Specifically, in the case of the Au_10_^2+^, eight electrons are distributed across over one S-MO and three P-MOs, while in the case of the Au_*x*_Pt_*y*_^2+^ clusters (*y* = 1–10) four electrons similarly delocalized over one S-MO and three P-MOs on each side (*cf.*Fig. 4, and S3–S14†). As the Pt size increases from *y* = 1 to *y* = 10, these beta *(10-1e)* bonds become impure due to a certain contamination with d-AOs of Pt atoms, as shown in Fig. S4–S14.† Nevertheless, the binary Au_*x*_Pt_*y*_^2+^ (*y* = 1–10) species still possess a (1S^2^1P^6^) shell and adopt a tetrahedral pyramid similar to that of the Au_10_^2+^, as depicted in Fig. S3–S14.† The preference for a tetrahedral shape in these binary dications can be attributed to the stability and electronic structure associated with this geometry. The tetrahedral arrangement allows for efficient bonding interactions between the Au and Pt atoms while maintaining the desired electronic configuration and stability for the clusters.

**Fig. 5:**
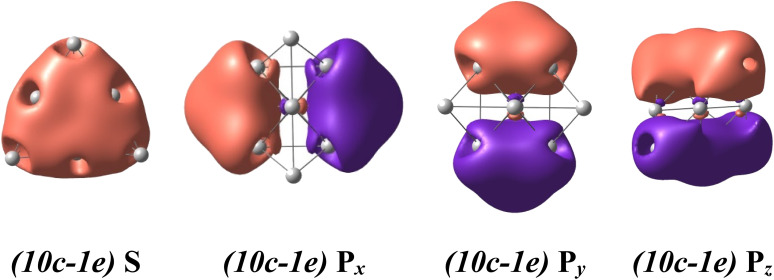
The metal aromaticity arising from *(10c-1e)* bonds of dicationic Au_*x*_Pt_*y*_^2+^ (y ranges from 1 to 10) clusters *via* AdNDP analysis at B3PW91/cc-pVTZ-PP theory level.

In addition to the prominent *(10c-1e)* bonds of spherical aromaticity, the Au_*x*_Pt_*y*_^2+^ (*y* = 1–10) clusters also display a variety of smaller multi-center bonds (*cf.*Fig. 5 and S4–S14†). These bonds result from the overlapping of equivalent atomic orbitals with similar symmetry and approximate energy levels of the d-electrons of Pt atoms. The presence and nature of these multi-center bonds depend on the specific positions of the Pt atoms within the cluster. By virtue of their spatial arrangement, these bonds contribute to the overall bonding pattern and electronic structure of the Au_*x*_Pt_*y*_^2+^ clusters. The formation of these multi-center bonds adds further complexity to the bonding interactions and highlights the role of Pt d-electrons in influencing the properties of these clusters.

**Fig. 6:**
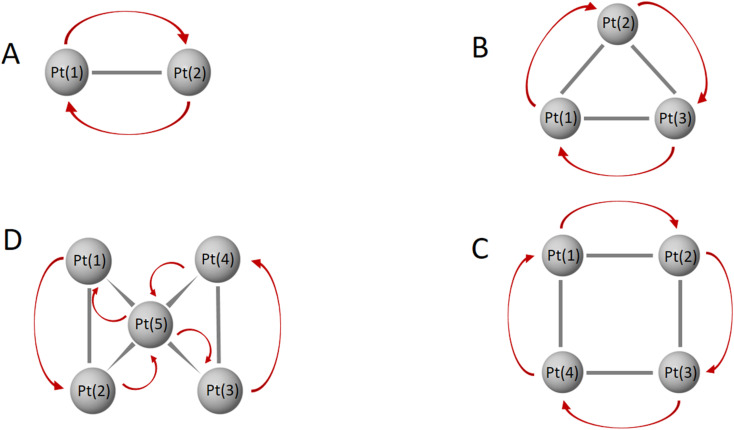
Electron movement among multi-center bonds of Pt atoms in the lowest-lying Au_*x*_Pt_*y*_^2+^ (*x* + *y* = 10) clusters with *y* = 2–5: Au_8_Pt_2_^2+^.A isomer (A), Au_7_Pt_3_^2+^.A isomer (B), Au_6_Pt_4_^2+^.A isomer (C), and Au_5_Pt_5_^2+^.A isomer (D).

The formation of bonds in Au_*x*_Pt_*y*_^2+^ is basically influenced by two trends based on their character, Au *vs.* Pt clusters, and thereby their structural tendencies with the critical point at *y* = 5. In the first trend which is observed for Au clusters (*y* ranging from 2 to 5), the Pt dopants tend to cluster together into symmetric blocks, with a preference for being located at the octahedral core. Since the number of d-electrons at these sizes is still small, one common d-electron is shared between two adjacent Pt dopants by an overlap of two equivalent atomic orbital having similar symmetry on the beta side to form one bond between two Pt centers *(2c-1e)* (*cf.*Fig. 6 and S5–S8†). In the second trend, with larger *y* values ranging from 5 to 10 giving rise to a series of Au-doped Pt clusters, the dominance and increased flexibility of the d-electrons on Pt atoms enable them to form numerous bonds that involve the delocalization of d electrons over more multiple centers, including *(3c-1e)* and *(6c-1e)*. This is in contrast to the Au clusters (with *y* < 6). The increase in the number of d-AOs provided by Pt atoms leads to significant contamination and heavier distortion of the S and P-MOs. (*cf.* Fig. S9–S14†).

In the Au_*x*_Pt_*y*_^2+^ clusters, it is observed in both alpha and beta side that each Au atom wholly retains five *(1c-1e)* electrons localized on its five d-orbitals, indicating that the d-electrons of Au atoms are not involved in muti-center bond formation (*cf.* Fig. S3–S14†). This contrasts with the behaviour of Pt atoms, whose beta d-electrons are more flexible and can participate in multi-center bonds, allowing them to connect with neighbouring Pt atoms. As a result, Pt atoms have a propensity to preferentially occupy the core of the binary clusters.

This discrepancy in behaviour between Au and Pt atoms can be attributed to the differences in their electronic structures and bonding capabilities. The localized nature of Au's d-electrons restricts their involvement in multi-center bonding, while the more flexible d-electrons of Pt enable them to engage in such bonding interactions. This distinction influences the preferred placement of Pt atoms in the core of the binary clusters, where they can effectively form multi-center bonds and establish connections with neighbouring Pt atoms.

In addition, Fig. S15† illustrates that the density of states (DOS) maps of the Au_*x*_Pt_*y*_^2+^ exhibit two distinct trends, which correspond to their respective structures with the critical point at *x* = *y* = 5, showing the structural impact on global population of electron. Notably, Au_10_^2+^ has the highest density of states, followed by a gradual decrease towards the Pt_10_^2+^. The DOS on the mixed Au_*x*_Pt_*y*_^2+^ is primarily contributed by the d-electrons of Au and Pt atoms, with Au making a more significant contribution. As the number of Au atoms in the cluster decreases from *x* = 10 to *x* = 0, there is a significant reduction in the DOS of Au_*x*_Pt_*y*_^2+^, especially from the critical point at *y* = 5 onward. Furthermore, the downward shift of the SOMO–LUMO gap on the beta side results in a reduced gap size (almost half) compared to the alpha side (see Table 2). This shift facilitates electron movement between the frontier orbital levels, ultimately causing an energetic degeneracy among the spin states within the clusters considered.

**Table:** Frontier orbital energy gap (eV) of the binary Au_*x*_Pt_*y*_^2+^ (*x* + *y* = 10, B3PW91/aug-cc-pVTZ-PP)

Clusters	Spin states	SOMO–LUMO (*α*)	SOMO–LUMO (*β*)
Au_10_^2+^	Singlet	3.88	
Au_9_Pt^2+^	Doublet	3.56	1.85
Au_8_Pt_2_^2+^	Triplet	3.55	1.46
Au_7_Pt_3_^2+^	Quartet	3.54	2.54
Au_6_Pt_4_^2+^	Quintet	3.46	2.15
Au_5_Pt_5_^2+^	Sextet	3.45	2.01
Au_4_Pt_6_^2+^	Septet	3.37	2.07
Au_3_Pt_7_^2+^	Octet	3.38	1.74
Au_2_Pt_8_^2+^	Nonet	3.47	1.95
AuPt_9_^2+^	Dectet	3.58	1.79
Pt_10_^2+^	11-et	3.52	1.82

## Concluding remarks

In the present theoretical study, the binary gold-platinum clusters Au_*x*_Pt_*y*_^2+^ containing ten atoms with *x* + *y* = 10 were systematically studied using DFT calculations with the B3PW91, TPSSh, revTPSS and BP86 functionals in conjunction with the aug-cc-pVTZ-PP basis set. The main results demonstrated that all the most stable structures of the Au_*x*_Pt_*y*_^2+^ clusters follow the tetrahedral pyramid pattern. In their dicationic state, a mixture of ten Au and Pt atoms consistently holds the pyramidal shape of both pure clusters. A critical point at *y* = 5 marks the onset of two distinct tendencies in the structural growth that characterize their chemical bonding.

For *y* = 2, 3, 4, and 5 where the Au_*x*_Pt_*y*_^2+^ are basically Au clusters, the Pt atoms gather together into symmetric Pt_*y*_ blocks as a line, a triangle, a quadrangle, and a tetragonal pyramid, respectively, located at the inner octahedral core of the cluster. At these sizes, some d-electrons of Pt atoms tend to form *(2c-1e)* bonds. Meanwhile, larger *y* = 6–10 leading to the doped Pt clusters, Pt atoms substitute Au atoms in the structure of the Au_5_Pt_5_^2+^.B isomer and form larger multi-center d-electron bonds.

All ten-atom binary Au_*x*_Pt_*y*_^2+^ clusters are characterized by bonds whose electrons are delocalized on S-MO and P-MOs for each alpha and beta stream. The S-MO and P-MOs are more distorted when the number of Pt atoms rise due to contaminations with d-AOs of Pt atoms. For the number of Pt atoms *y* = 1–6, the mixed clusters Au_*x*_Pt_*y*_^2+^ are less stable than the pure gold Au_10_^2+^ cluster (in terms of dissociation energy) but from *y* = 7 onwards, the system becomes Pt clusters, and they can be more stabilized by the formation of the stronger Pt–Pt bonds. In fact, the pure platinum Pt_10_^2+^, corresponding to *y* = 10, exhibits the highest thermodynamic stability. Furthermore, the substitution of Pt atoms into the pure Au_10_^2+^ cluster increases significantly the magnetic property. The spin density of the Au_*x*_Pt_*y*_^2+^ clusters is mainly located on Pt atoms and the total magnetic moment increase steadily from 0 to 10 *μ*_B_, corresponding to the number *y* of Pt atoms increased from 0 to 10 with calculations using B3PW91 and TPSSh functionals. Overall, admixture of both Au and Pt atoms within a small size emerges as an elegant way of keeping the small pyramidal structure but bringing in a high and controllable magnetic moment.

## Conflicts of interest

The authors declare no competing financial interest.

## Supplementary Material

RA-013-D3RA06000D-s001
