# Supplementary material for: Formation of pyramidal structures through mixing gold and platinum atoms: the AuxPty2+ clusters with x + y = 10
Source: RSC Adv. 2023 Nov 8;13(47):32893–903. doi: 10.1039/d3ra06000d (PMC10630918; doi:10.1039/d3ra06000d)
Supplement: RA-013-D3RA06000D-s001 [file RA-013-D3RA06000D-s001.pdf]

Electronic Supplementary Material (ESI) for RSC Advances.
This journal is © The Royal Society of Chemistry 2023

Supplementary Information (SI) File

Formation of pyramidal structures through mixing gold and platinum atoms: The $Au_xPt_y^{2+}$ clusters with x + y = 10

Bao-Ngan Nguyen-Ha,[1,2] Cam-Tu Phan Dang,[3,4] Long Van Duong,[2,5] My Phuong Pham-Ho,[6,7] Minh Tho Nguyen,[1,2,*] Nguyen Minh Tam[8,*]

[1] Laboratory for Chemical Computation and Modeling, Institute for Computational Science and Artificial Intelligence, Van Lang University, Ho Chi Minh City, Vietnam. Email: ngan.nguyenhabao@vlu.edu.vn; minhtho.nguyen@vlu.edu.vn

[2] Faculty of Applied Technology, School of Technology, Van Lang University, Ho Chi Minh City, Vietnam

[3] Faculty of Natural Sciences, Duy Tan University, Da Nang, Vietnam

[4] Institute of Research and Development, Duy Tan University, Da Nang, Vietnam

[5] Atomic Molecular and Optical Physics Research Group, Science and Technology Advanced Institute, Van Lang University, Ho Chi Minh City, Vietnam

[6] Faculty of Chemical Engineering, Ho Chi Minh City University of Technology (HCMUT), 268 Ly Thuong Kiet Street, District 10, Ho Chi Minh City, Vietnam

[7] Vietnam National University Ho Chi Minh City, Linh Trung Ward, Thu Duc City, Ho Chi Minh City, Vietnam.

[8] Faculty of Basic Sciences, University of Phan Thiet, 225 Nguyen Thong, Phan Thiet City, Binh Thuan, Vietnam. Email: nmtam@upt.edu.vn

Contents:

i) Structures, relative energies (eV), and spin states (in bracket) of the low-lying isomers of $Au_xPt_y^{2+}$ with x + y =10) using B3PW91, TPSSh, BP86 and revTPSS/ aug-cc-pVTZ-PP + ZPE computations. (Figure S1 and S2)

ii) Adaptive natural density partitioning (AdNDP) analysis of the $Au_xPt_y^{2+}$ clusters with x + y = 10 at B3PW91/ cc-pVTZ-PP theory level (Figures S3-S14).

iii) Calculated density of states (DOS) of the $Au_xPt_y^{2+}$ (x + y = 10) at B3PW91/aug-cc-pVTZ-PP theory level (Figure S15).

iv) Optimized geometrical shapes and Cartesian coordinates of the lowest-lying $Au_xPt_y^{2+}$ (x + y = 10) isomers calculated at B3PW91/ aug-cc-pVTZ-PP method (Table S1).

Method	Structures			
	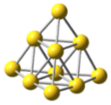 **Au_{10}^{2+}** (singlet)			
	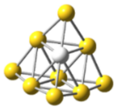 **Au_9Pt^{2+}.A**	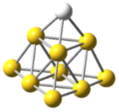 **Au_9Pt^{2+}.B**	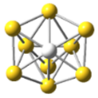 **Au_9Pt^{2+}.C**	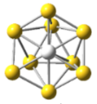 **Au_9Pt^{2+}.D**
B3PW91	0.00 (doublet)	0.42 (doublet)	0.42 (doublet)	0.47 (doublet)
TPSSh	0.00 (doublet)	0.42 (doublet)	0.37 (doublet)	0.30 (doublet)
BP86	0.00 (doublet)	0.31 (doublet)	0.48 (doublet)	0.49 (doublet)
revTPSS	0.00 (doublet)	0.37 (doublet)	0.35 (doublet)	0.26 (doublet)
	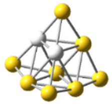 **$Au_8Pt_2^{2+}$.A**	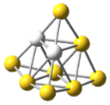 **$Au_8Pt_2^{2+}$.A**	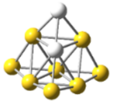 **$Au_8Pt_2^{2+}$.B**	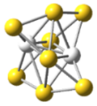 **$Au_8Pt_2^{2+}$.D**
B3PW91	0.00 (triplet)	0.07 (singlet)	0.17 (triplet)	0.18 (singlet)
TPSSh	0.00 (triplet)	0.11 (singlet)	0.16 (triplet)	0.20 (triplet)
BP86	0.00 (triplet)	0.10 (singlet)	0.15 (triplet)	0.13 (triplet)
revTPSS	0.00 (triplet)	0.12 (singlet)	0.15 (triplet)	0.24 (triplet)
	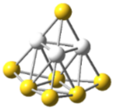 **$Au_7Pt_3^{2+}$.A**	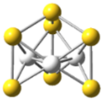 **$Au_7Pt_3^{2+}$.B**	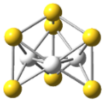 **$Au_7Pt_3^{2+}$.B**	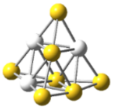 **$Au_7Pt_3^{2+}$.C**
B3PW91	0.00 (quartet)		0.52 (quartet)	0.52 (quartet)
TPSSh	0.00 (quartet)	0.22 (doublet)	0.49 (quartet)	0.44 (quartet)
BP86	0.00 (quartet)	0.16 (doublet)	0.37 (quartet)	0.39 (quartet)
revTPSS	0.00 (quartet)		0.42 (quartet)	0.38 (quartet)
	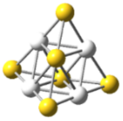 **$Au_6Pt_4^{2+}$.A**	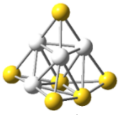 **$Au_6Pt_4^{2+}$.B**	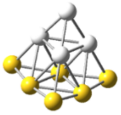 **$Au_6Pt_4^{2+}$.C**	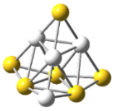 **$Au_6Pt_4^{2+}$.D**
B3PW91	0.00 (quintet)	0.17 (quintet)	0.34 (quintet)	0.46 (quintet)
TPSSh	0.00 (quintet)	0.17 (quintet)	0.26 (quintet)	0.44 (quintet)
BP86	0.00 (quintet)	0.17 (quintet)	0.04 (quintet)	0.25 (quintet)
revTPSS	0.00 (quintet)	0.16 (quintet)	0.09 (quintet)	0.33 (quintet)
	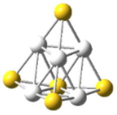 **$Au_5Pt_5^{2+}$.A**	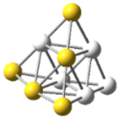 **$Au_5Pt_5^{2+}$.B**	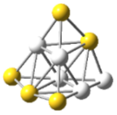 **$Au_5Pt_5^{2+}$.C**	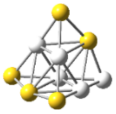 **$Au_5Pt_5^{2+}$.C**
B3PW91	0.00 (sextet)	0.11 (sextet)	0.15 (quartet)	0.17 (sextet)
TPSSh	0.00 (sextet)	0.14 (sextet)	0.13 (quartet)	0.15 (sextet)
BP86	0.03 (sextet)	0.05 (sextet)	0.00 (quartet)	0.05 (sextet)
revTPSS	0.00 (sextet)	0.02 (sextet)	0.03 (quartet)	0.08 (sextet)
CCSD(T) (*)	0.00 (sextet)	0.13 (sextet)		

Figure S1. Structures, relative energies (eV), and spin states (in bracket) of the most stable isomers of $Au_xPt_y^{2+}$ with x + y =10 and y = 0 – 5) using B3PW91, TPSSh, BP86 and revTPSS/ aug-cc-pVTZ-PP + ZPE computations. (*) is calculated at CCSD(T)/ cc-pVDZ-PP method. Yellow ball = Au and white ball = Pt.

Method	Structures			
	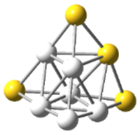<br>$Au_4Pt_6^{2+}$.A/L	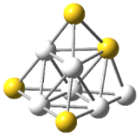<br>$Au_4Pt_6^{2+}$.B	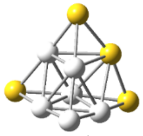<br>$Au_4Pt_6^{2+}$.A	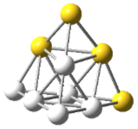<br>$Au_4Pt_6^{2+}$.D
B3PW91	0.00 (septet)	0.27 (septet)	0.48 (quintet)	0.61 (quintet)
TPSSh	0.00 (septet)	0.28 (septet)	0.20 (quintet)	0.55 (quintet)
BP86	0.00 (septet)	0.22 (septet)	0.12 (quintet)	0.25 (quintet)
revTPSS	0.00 (septet)	0.26 (septet)	0.17 (quintet)	0.55 (quintet)
	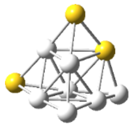<br>$Au_3Pt_7^{2+}$.A/L	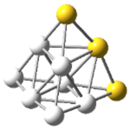<br>$Au_3Pt_7^{2+}$.B	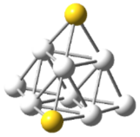<br>$Au_2Pt_8^{2+}$.A	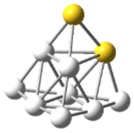<br>$Au_2Pt_8^{2+}$.B
B3PW91	0.00 (octet)<br>0.10 (sextet)<br>0.28 (quartet)	0.11 (octet)	0.00 (nonet)<br>0.36 (septet)	0.56 (nonet)<br>0.64 (septet)
TPSSh	0.01 (octet)	0.00 (sextet)<br>0.05 (octet)	0.00 (nonet)<br>0.35 (septet)	0.59 (nonet)<br>0.56 (septet)
BP86	0.09 (sextet)<br>0.10 (octet)	0.00 (sextet)<br>0.04 (octet)	0.00 (nonet)	0.08 (quintet)<br>0.16 (septet)
revTPSS	0.10 (octet)<br>0.13 (sextet)<br>0.28 (quartet)	0.00 (sextet)<br>0.06 (octet)	0.00 (nonet)<br>0.28 (septet)	0.26 (quintet)<br>0.42 (nonet)
	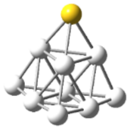<br>$AuPt_9^{2+}$.A	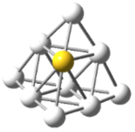<br>$AuPt_9^{2+}$.B	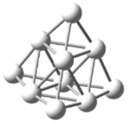<br>Pt_{10}^{2+}	
B3PW91	0.00 (10-et)<br>0.14 (octet)<br>0.17 (sextet)	0.80 (quartet)	0.00 (11-et)<br>0.10 (nonet)<br>0.30 (septet)	
TPSSh	0.00 (10-et)<br>0.07 (octet)<br>0.12 (sextet)	0.62 (quartet)	0.00 (11-et)<br>0.07 (nonet)<br>0.04 (septet)	
BP86	0.00 (octet)<br>0.05 (sextet)<br>0.11 (10-et)	0.13 (quartet)	0.00 (septet)<br>0.00 (quintet)<br>0.30 (nonet)<br>0.30 (11-et)	
revTPSS	0.07 (10-et)<br>0.00 (octet)<br>0.07 (sextet)	0.29 (quartet)	0.40 (11-et)<br>0.26 (nonet)<br>0.00 (septet)	

Figure S2. Structures, relative energies (eV), and spin states (in bracket) of the most stable isomers of $Au_xPt_y^{2+}$ with x + y =10 and y = 6 - 10) using B3PW91, TPSSh, BP86 and revTPSS/ aug-cc-pVTZ-PP + ZPE computations. Yellow ball = Au and white ball = Pt.

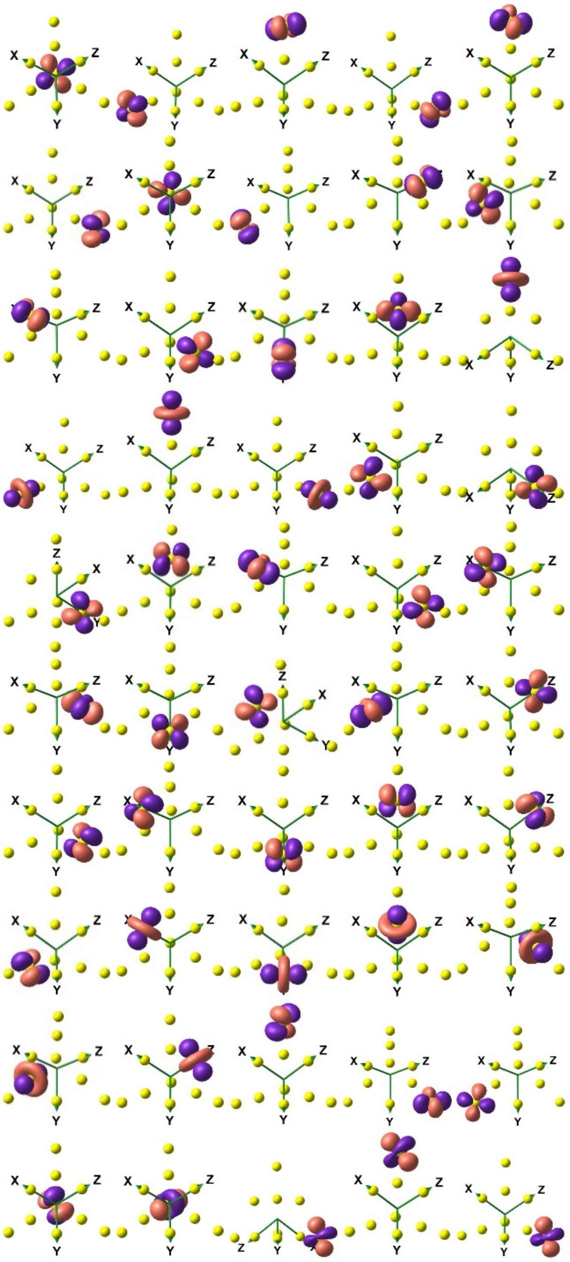

50 x *(2c-1e)*

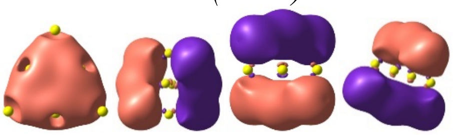

4 x *(10c-2e)*

Figure S3: AdNDP analysis showing muti-center bonds in the $\mathbf{Au_{10}}^{2+}$ cluster at singlet state, calculated at B3PW91/ cc-pVTZ-PP theory level.

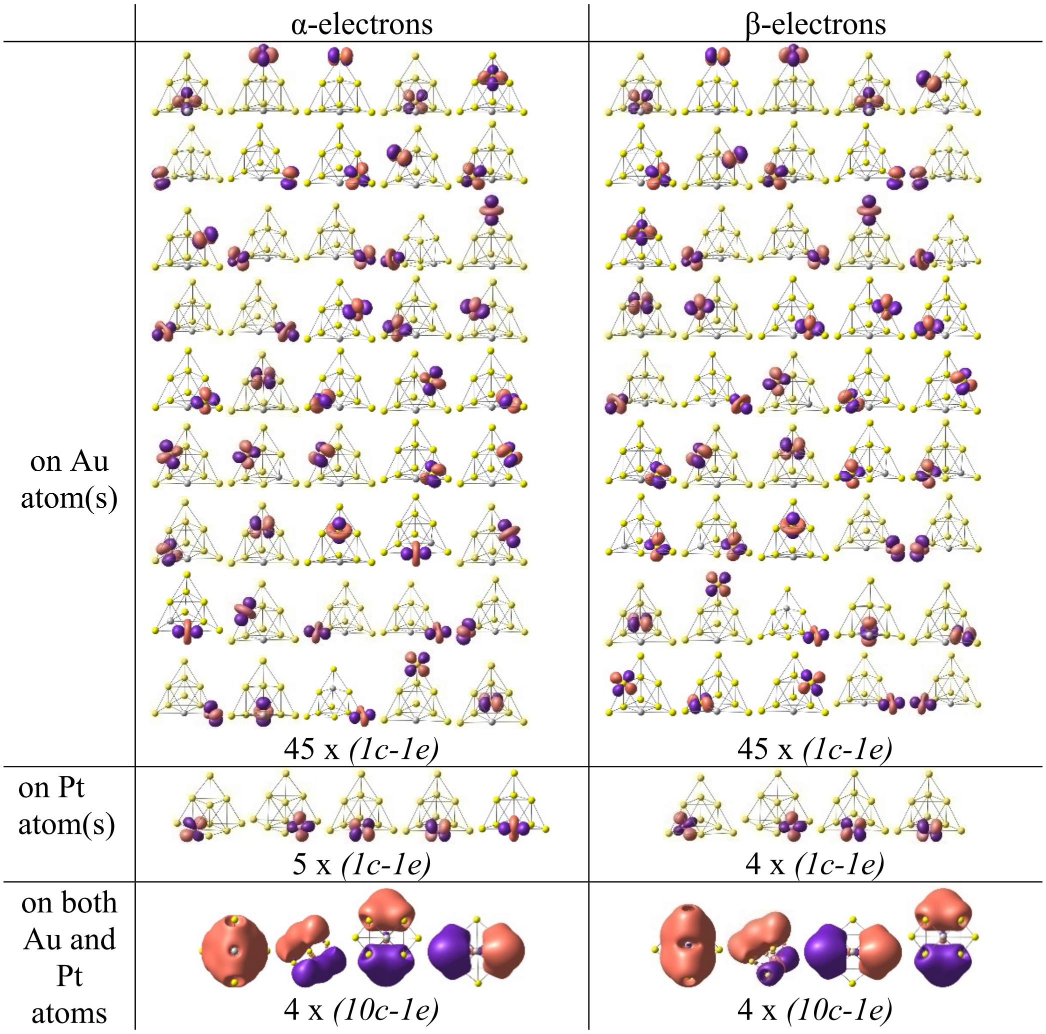

Figure S4: AdNDP analysis showing muti-center bonds in the $\mathbf{Au_9Pt^{2+}.A}$ cluster at doublet state, calculated at B3PW91/ cc-pVTZ-PP theory level.

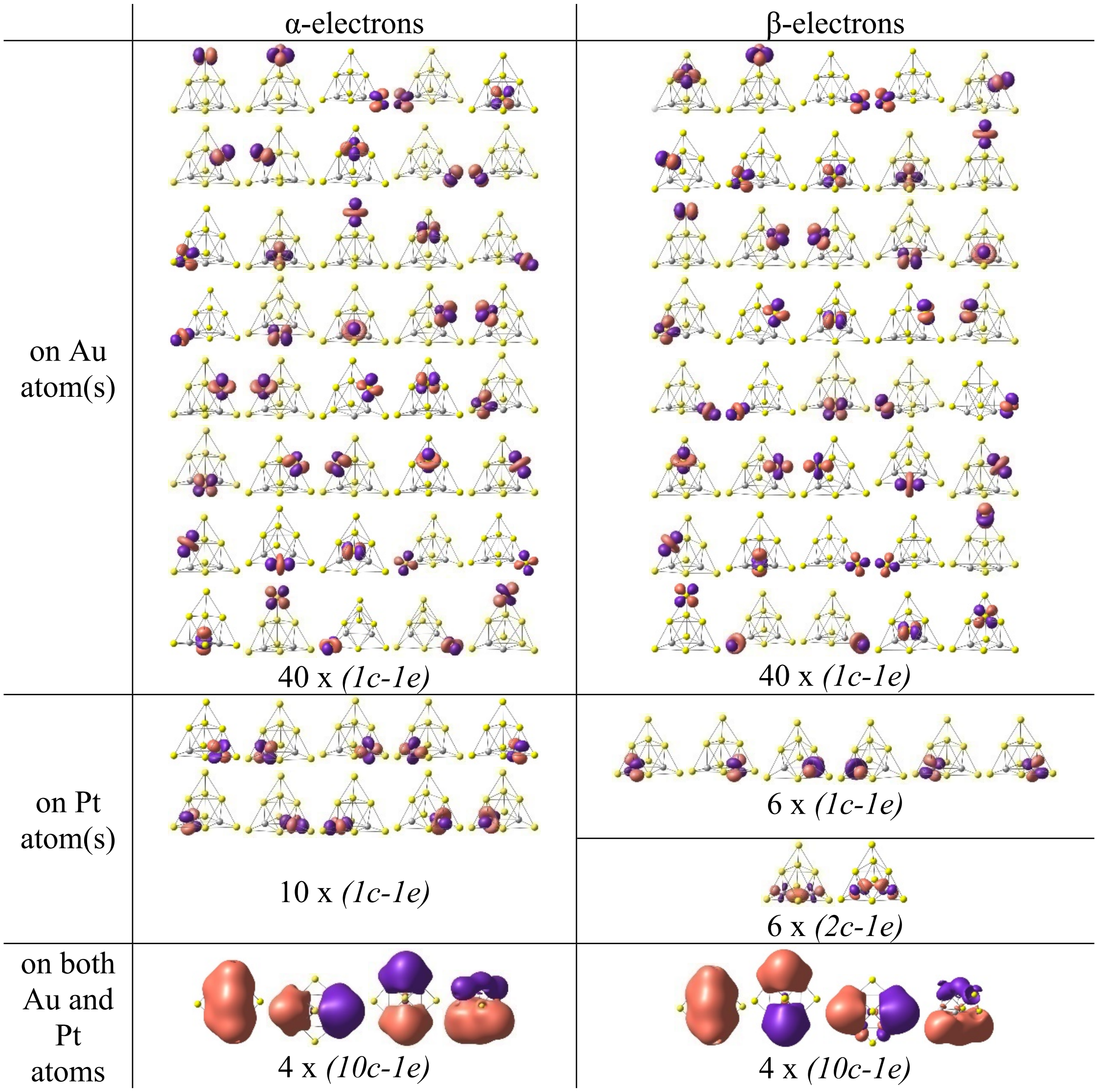

Figure S5: AdNDP analysis showing muti-center bonds in the **$Au_8Pt_2^{2+}$.A** cluster at triplet state, calculated at B3PW91/ cc-pVTZ-PP theory level.

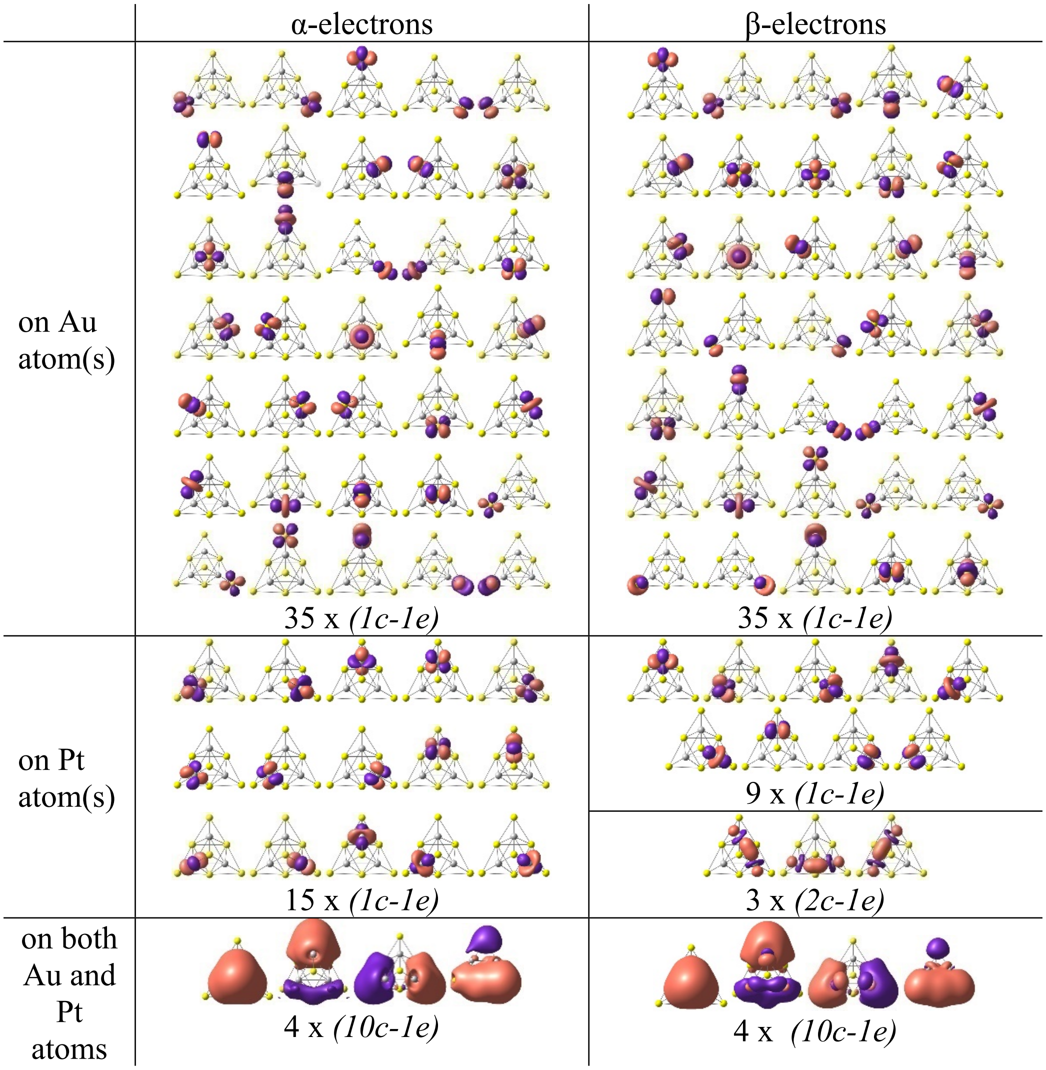

Figure S6: AdNDP analysis showing muti-center bonds in the $\mathbf{Au_7Pt_3^{2+}}$**.A** cluster at quartet state, calculated at B3PW91/ cc-pVTZ-PP theory level.

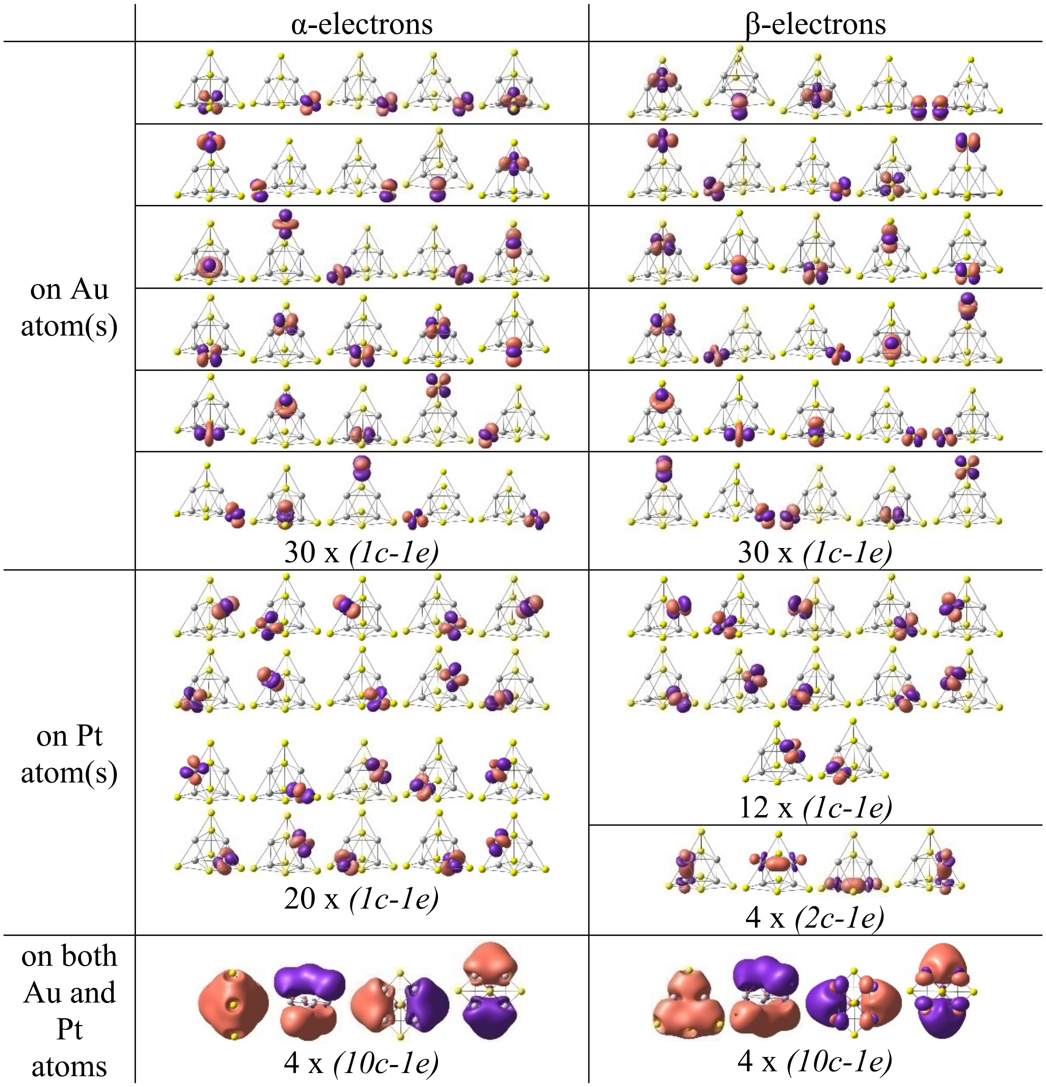

Figure S7: AdNDP analysis showing muti-center bonds in the $\mathbf{Au_6Pt_4^{2+}.A}$ cluster at quintet state, calculated at B3PW91/ cc-pVTZ-PP theory level.

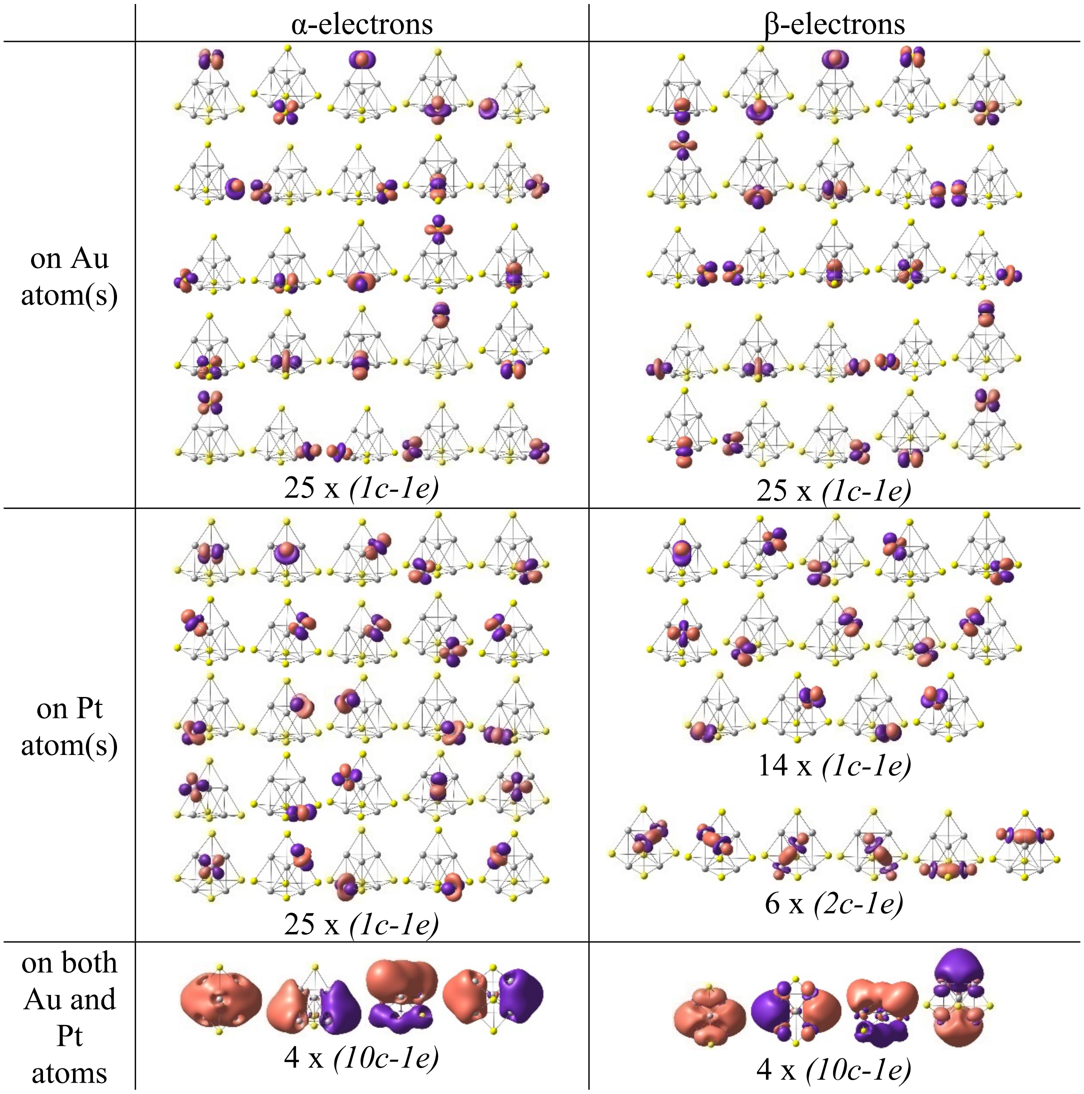

Figure S8: AdNDP analysis showing muti-center bonds in the $\mathbf{Au_5Pt_5^{2+}}$**.A** cluster at sextet state, calculated at B3PW91/ cc-pVTZ-PP theory level.

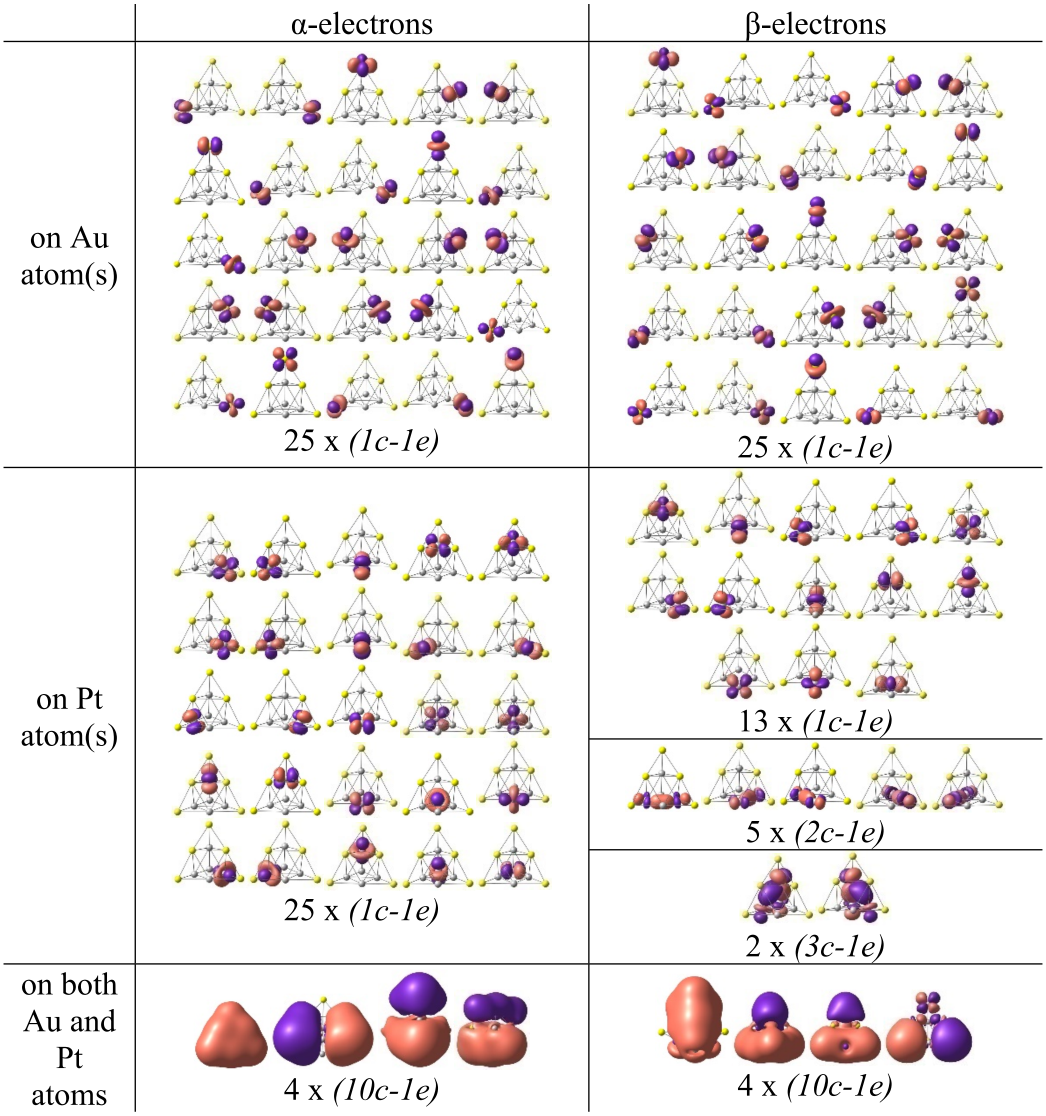

Figure S9: AdNDP analysis showing muti-center bonds in the $\mathbf{Au_5Pt_5^{2+}.B}$ cluster at sextet state, calculated at B3PW91/ cc-pVTZ-PP theory level.

	α-electrons	β-electrons

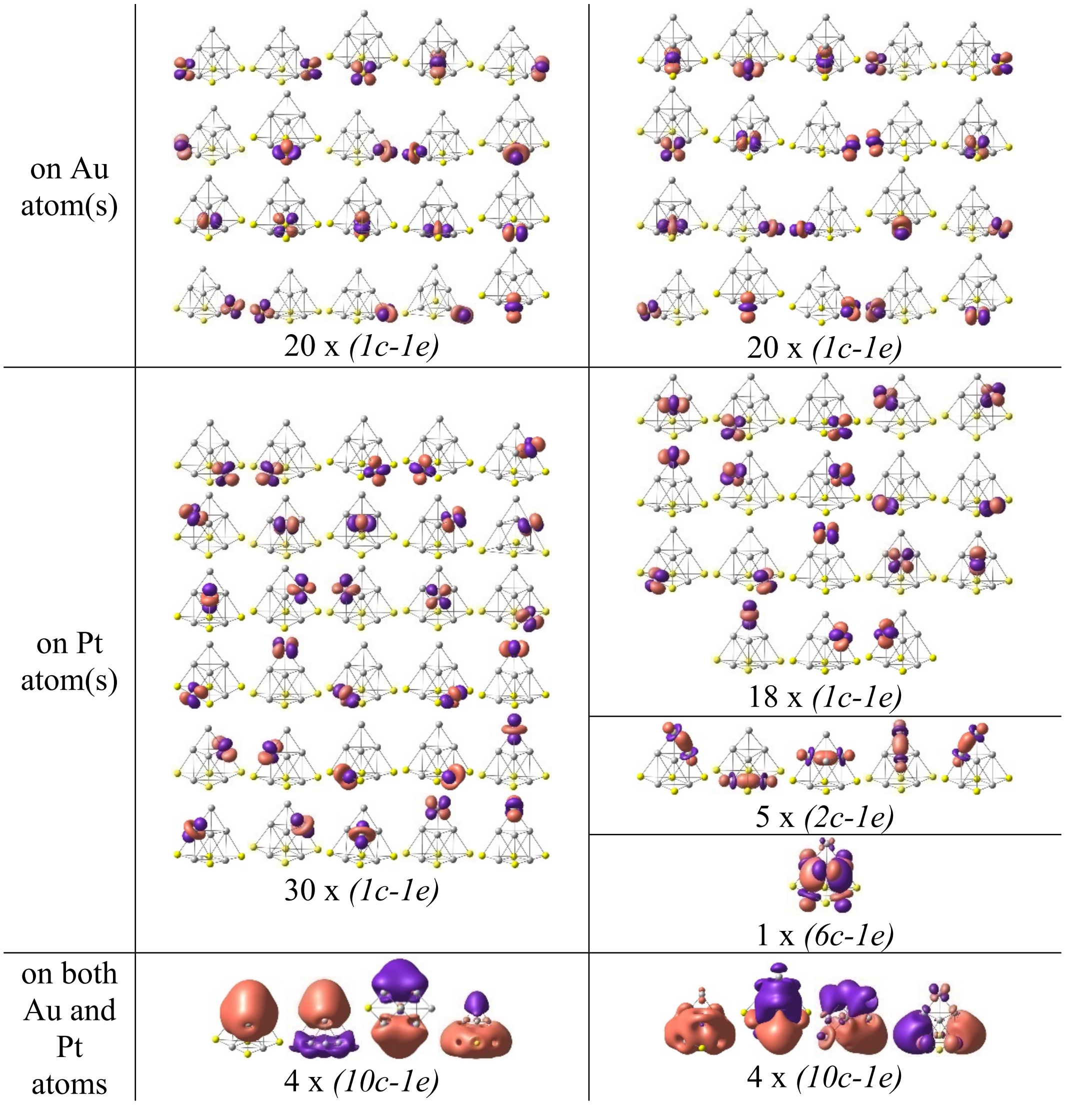

Figure S10: AdNDP analysis showing muti-center bonds in the $\mathbf{Au_4Pt_6^{2+}.A}$ cluster at septet state, calculated at B3PW91/ cc-pVTZ-PP theory level.

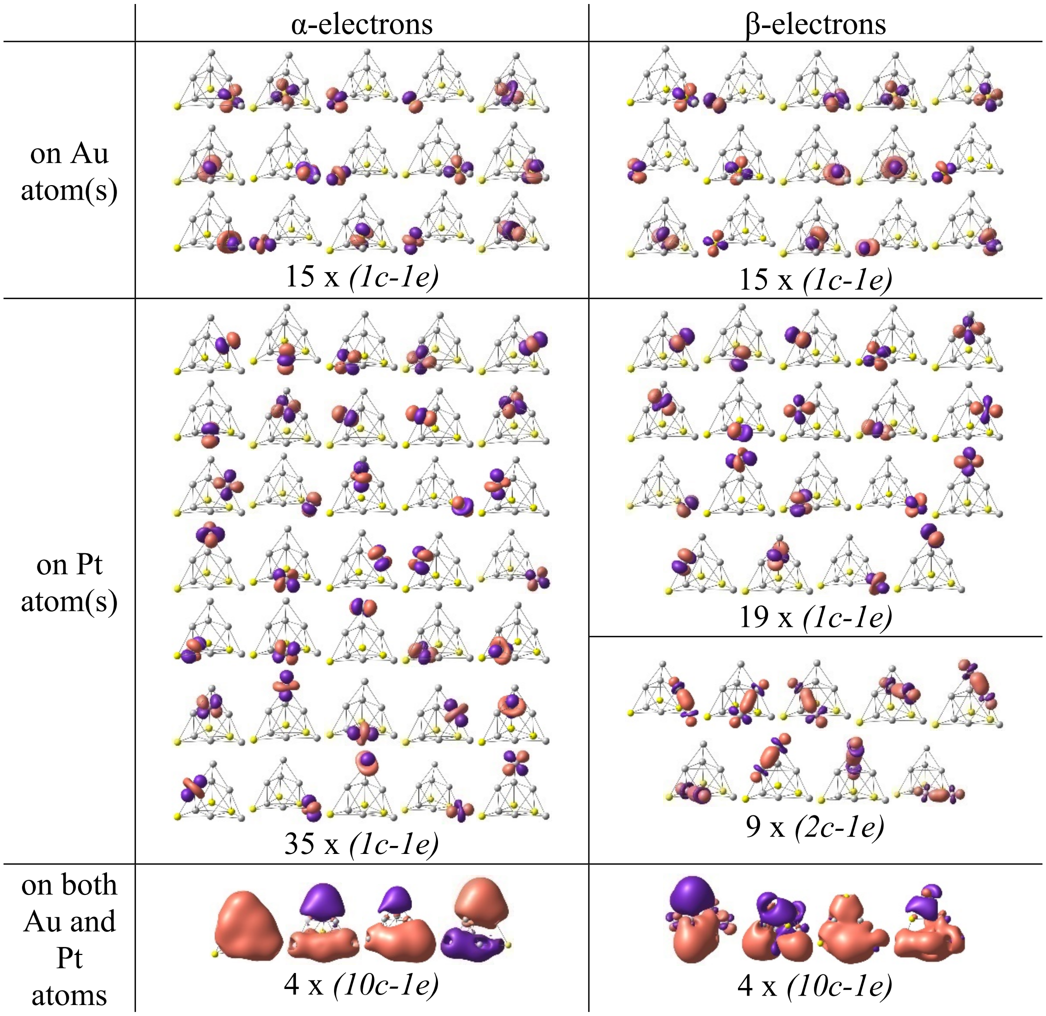

Figure S11: AdNDP analysis showing muti-center bonds in the $\mathbf{Au_3Pt_7^{2+}}$**.A** cluster at octet state, calculated at B3PW91/ cc-pVTZ-PP theory level.

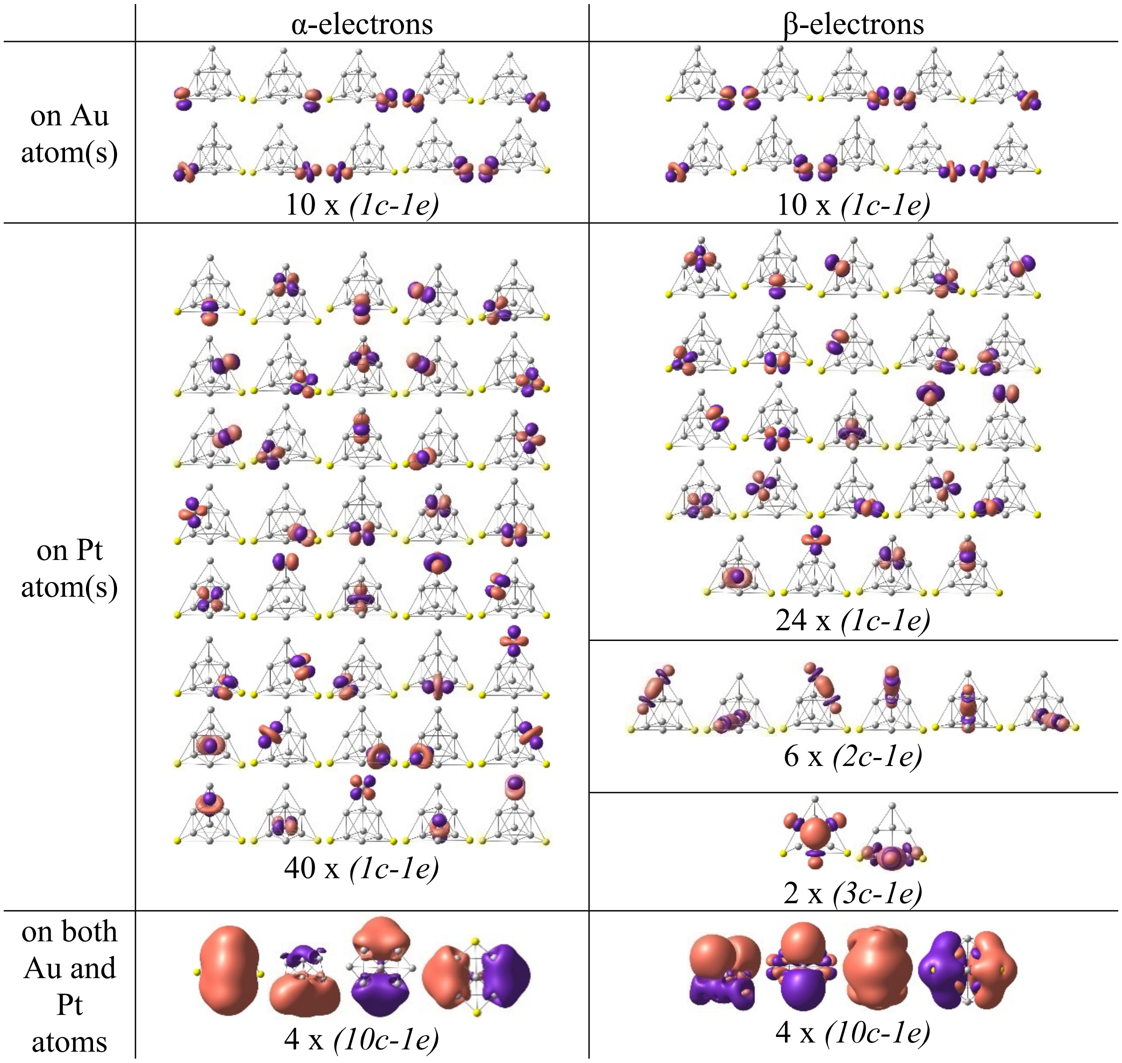

Figure S12: AdNDP analysis showing muti-center bonds in the $\mathbf{Au_2Pt_8^{2+}.A}$ cluster at nonet state, calculated at B3PW91/ cc-pVTZ-PP theory level.

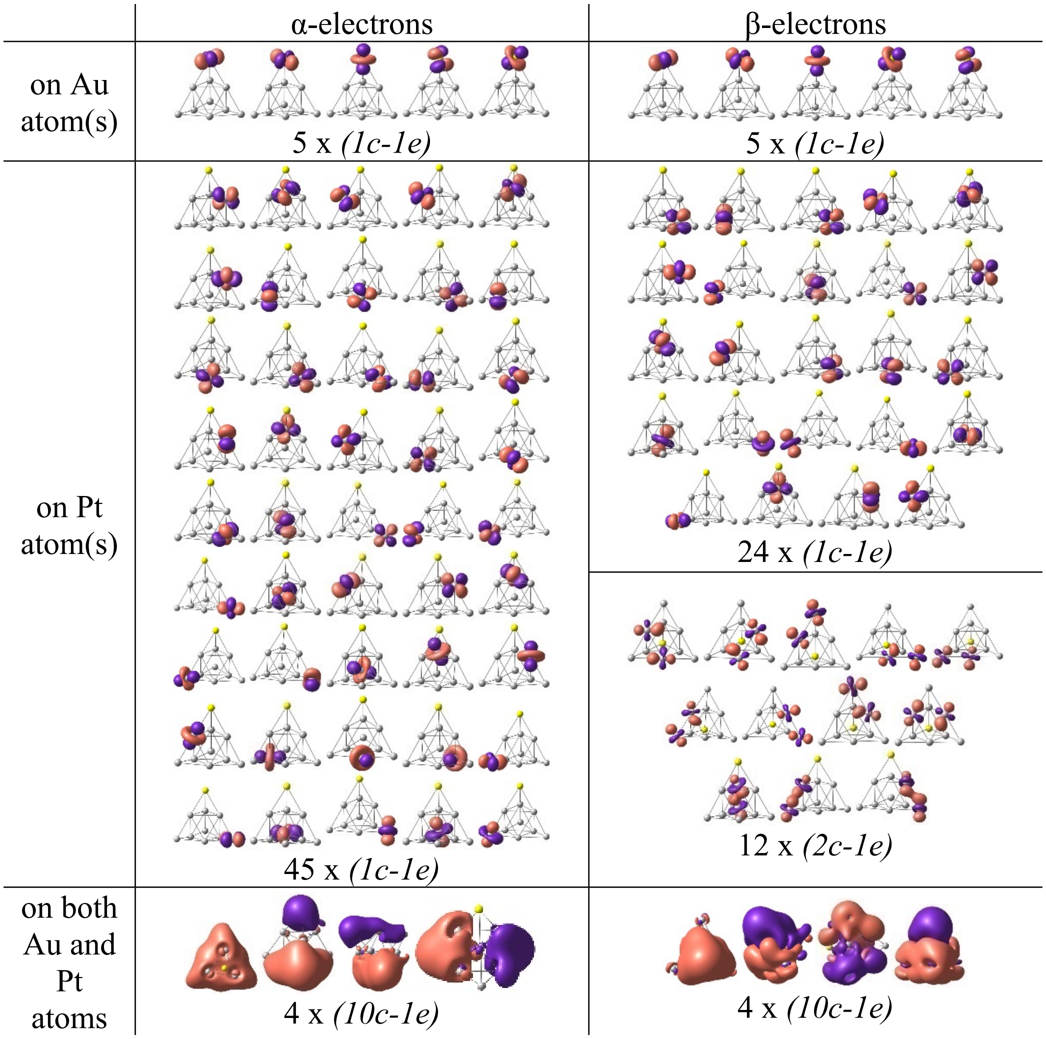

Figure S13: AdNDP analysis showing muti-center bonds in the $\mathbf{AuPt_9^{2+}.A}$ cluster at 10-et state, calculated at B3PW91/ cc-pVTZ-PP theory level.

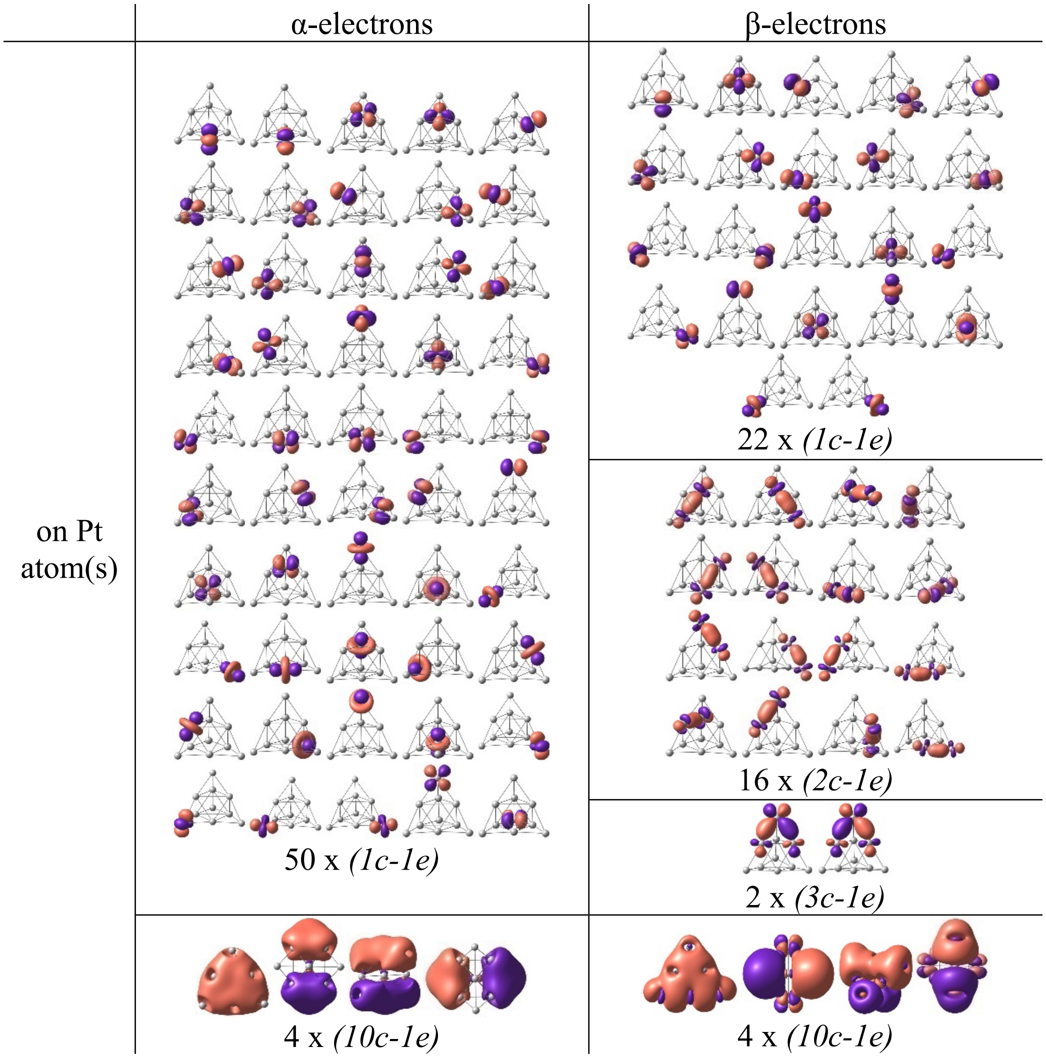

Figure S14: AdNDP analysis showing muti-center bonds in the $\mathbf{Pt_{10}^{2+}.A}$ cluster at 11-et state, calculated at B3PW91/ cc-pVTZ-PP theory level.

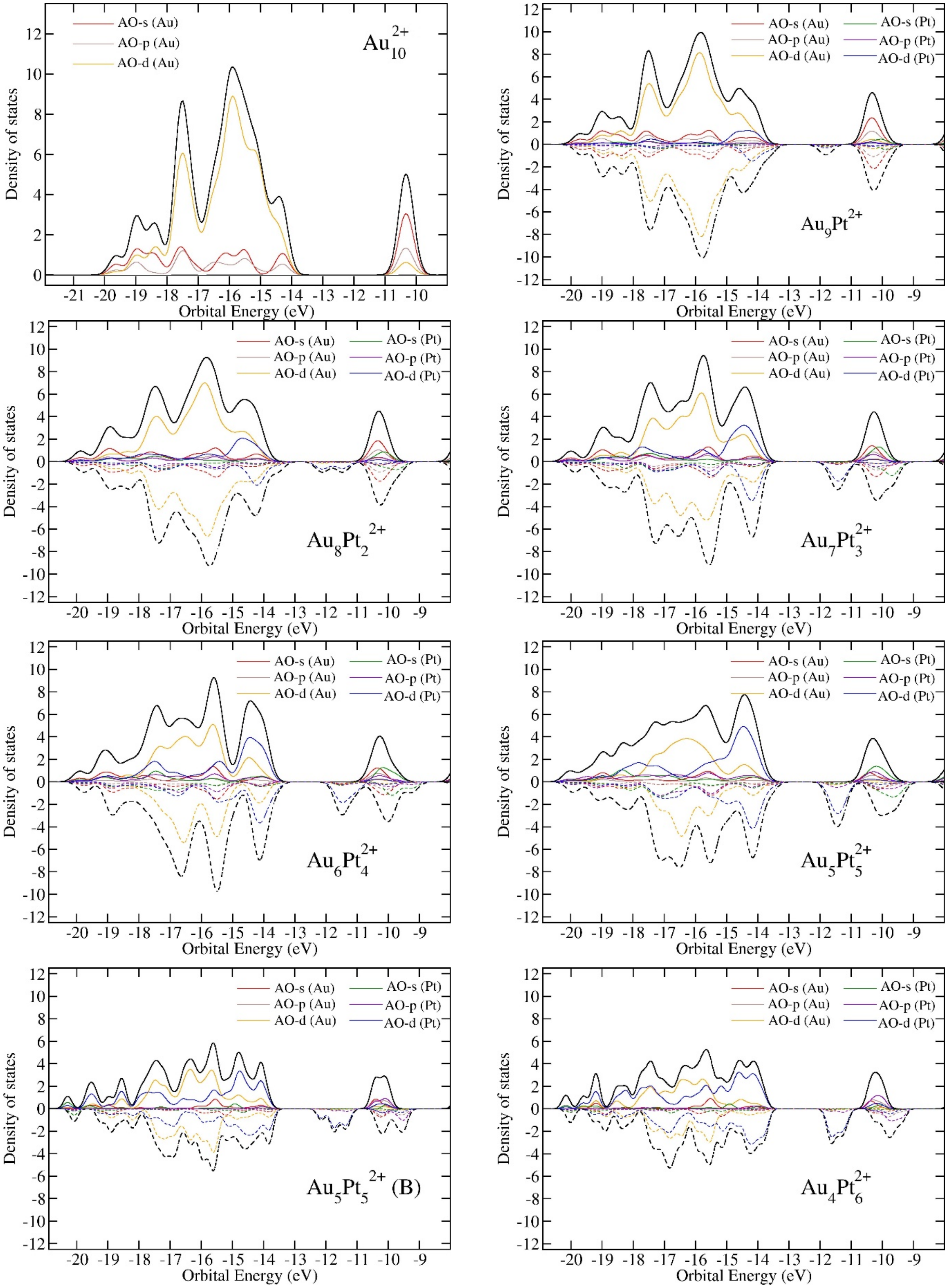

AO-s (Au)
AO-p (Au)
AO-d (Au)
AO-s (Pt)
AO-p (Pt)
AO-d (Pt)
Au_{10}^{2+}
Au_9Pt^{2+}
$Au_8Pt_2^{2+}$
$Au_7Pt_3^{2+}$
$Au_6Pt_4^{2+}$
$Au_5Pt_5^{2+}$
$Au_5Pt_5^{2+}$ (B)
$Au_4Pt_6^{2+}$
Density of states
Orbital Energy (eV)

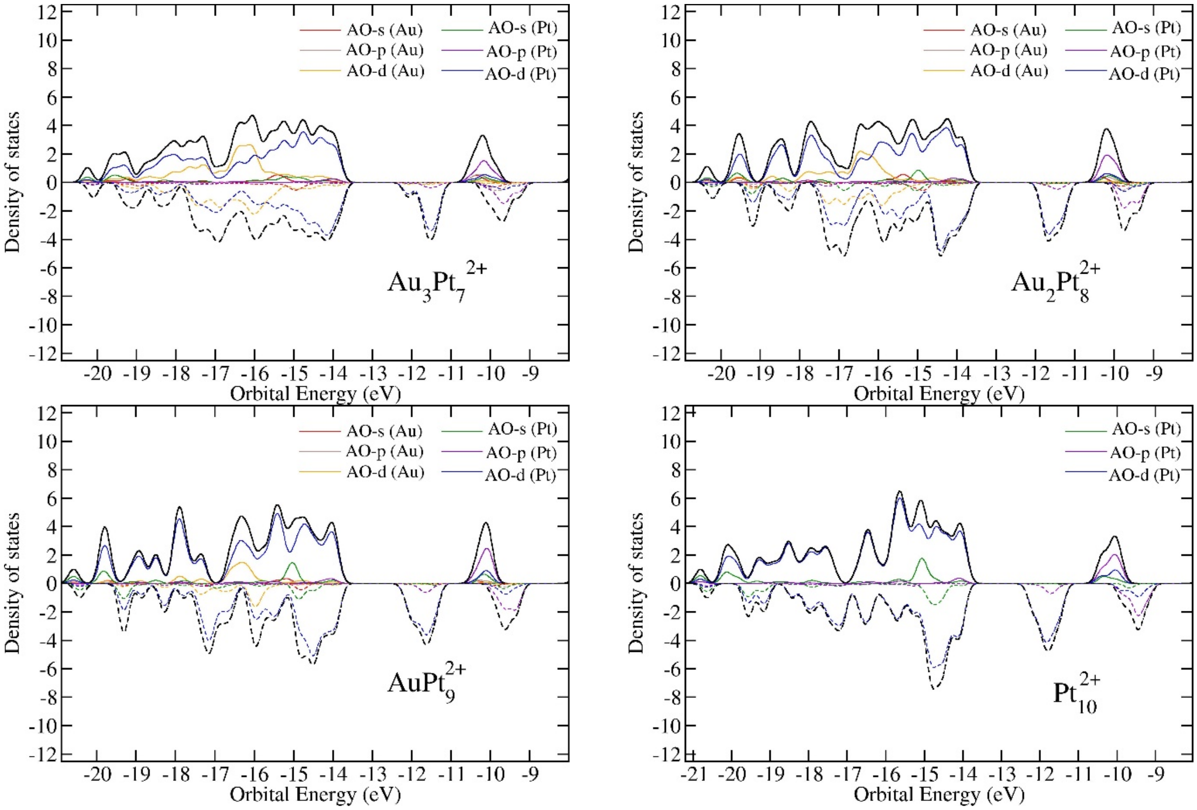

Figure S15: Calculated density of states (DOS) of $\mathbf{Au_xPt_y}^{2+}$ (x+y = 10) at B3PW91/aug-cc-pVTZ-PP level. Positive and negative DOS represent spin-up and spin-down electrons, respectively.

Table S1: Optimized geometrical shapes and Cartesian coordinates of the lowest-lying $Au_xPt_y^{2+}$ (x + y = 10) isomers calculated at B3PW91/aug-cc-pVTZ-PP method.

Isomer	Coordinates			
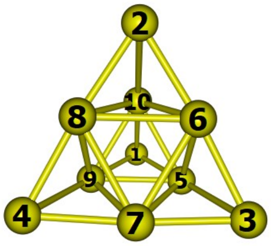 Au_{10}^{2+} (T_d) singlet	Au	-1.90919614	1.90919614	1.90919614
	Au	1.90919614	-1.90919614	1.90919614
	Au	1.90919614	1.90919614	-1.90919614
	Au	-1.90919614	-1.90919614	-1.90919614
	Au	-0.00000000	2.02642215	0.00000000
	Au	2.02642215	-0.00000000	0.00000000
	Au	-0.00000000	0.00000000	-2.02642215
	Au	0.00000000	-2.02642215	-0.00000000
	Au	-2.02642215	0.00000000	0.00000000
	Au	0.00000000	-0.00000000	2.02642215
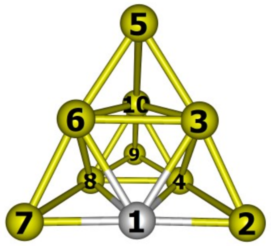 Au_9Pt^{2+}.A (C_{2v}) doublet	Pt	0.00000000	-0.00000000	-1.90497415
	Au	0.00000000	2.63733420	-1.95262215
	Au	1.45503911	1.44306111	-0.00612800
	Au	-1.45503911	1.44306111	-0.00612800
	Au	2.70442921	-0.00000000	1.90662515
	Au	1.45503911	-1.44306111	-0.00612800
	Au	0.00000000	-2.63733420	-1.95262215
	Au	-1.45503911	-1.44306111	-0.00612800
	Au	-2.70442921	0.00000000	1.90662515
	Au	-0.00000000	0.00000000	1.99736915
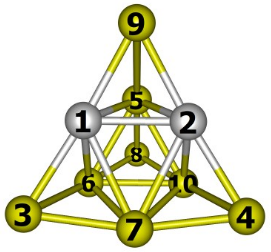 $Au_8Pt_2^{2+}$.A (C_s) triplet	Pt	1.14144281	0.81770330	1.25838410
	Pt	1.14144281	0.81770330	-1.25838410
	Au	1.14144281	-1.44589787	2.68187220
	Au	1.14144281	-1.44589787	-2.68187220
	Au	-1.15710836	1.63808536	0.00000000
	Au	-1.18545936	-0.86639583	1.42614911
	Au	1.16466782	-1.76958090	0.00000000
	Au	-3.31907953	-0.02144677	0.00000000
	Au	1.14556481	3.16282448	0.00000000
	Au	-1.18545936	-0.86639583	-1.42614911
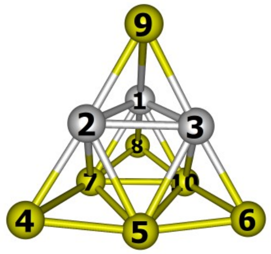 $Au_7Pt_3^{2+}$.A (C_{3v}) quartet	Pt	1.29498210	-0.74765806	1.14725809
	Pt	-0.00000009	1.49531657	1.14725809
	Pt	-1.29498201	-0.74765822	1.14725809
	Au	0.00000000	3.10231624	-0.99291807
	Au	-1.44459311	0.83403606	-1.25428709
	Au	-2.68668459	-1.55115797	-0.99291807
	Au	1.44459289	0.83403645	-1.25428709
	Au	2.68668459	-1.55115797	-0.99291807
	Au	0.00000000	0.00000010	3.34340826
	Au	0.00000022	-1.66807222	-1.25428709

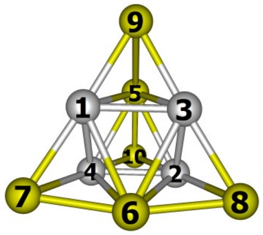

$Au_6Pt_4^{2+}$.A
(D_{2d})
quintet

Pt	1.29483810	1.29483810	0.00000000
Pt	-1.29483810	-1.29483810	0.00000000
Pt	-1.29483810	1.29483810	0.00000000
Pt	1.29483810	-1.29483810	0.00000000
Au	0.00000000	0.00000000	2.26016317
Au	0.00000000	0.00000000	-2.26016317
Au	2.66886321	0.00000000	-1.87859514
Au	-2.66886321	0.00000000	-1.87859514
Au	0.00000000	2.66886321	1.87859514
Au	0.00000000	-2.66886321	1.87859514

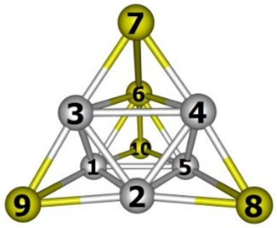

$Au_5Pt_5^{2+}$.A
(C_{2v})
sextet

Pt	-1.52136112	1.29029010	-0.01516100
Pt	0.00000000	0.00000000	-1.65814813
Pt	-1.52136112	-1.29029010	-0.01516100
Pt	1.52136112	-1.29029010	-0.01516100
Pt	1.52136112	1.29029010	-0.01516100
Au	0.00000000	0.00000000	2.09950616
Au	-0.00000000	-2.66868620	1.69694013
Au	2.83403722	-0.00000000	-1.89817514
Au	-2.83403722	0.00000000	-1.89817514
Au	0.00000000	2.66868620	1.69694013

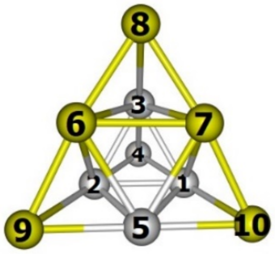

$Au_5Pt_5^{2+}$.B
(C_s)
sextet

Pt	0.01264400	1.39250311	1.29322510
Pt	0.01264400	1.39250311	-1.29322510
Pt	-1.88017814	0.09212801	0.00000000
Pt	-1.79734514	2.65057320	-0.00000000
Pt	1.86005114	0.10289601	-0.00000000
Au	0.01264400	-1.52859812	-1.44141611
Au	0.01264400	-1.52859812	1.44141611
Au	-2.00763016	-2.60502820	0.00000000
Au	1.87591914	0.05144700	-2.67775920
Au	1.87591914	0.05144700	2.67775920

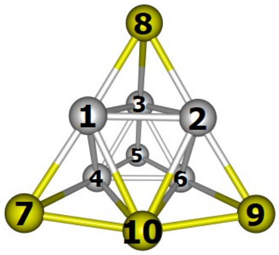

$Au_4Pt_6^{2+}$.A
(C_s)
septet

Pt	1.08191108	-0.79568106	1.29798410
Pt	1.08191108	-0.79568106	-1.29798410
Pt	1.14878909	1.62064913	0.00000000
Pt	-1.06880508	0.85748406	1.31265310
Pt	-0.93999007	3.02964323	0.00000000
Pt	-1.06880508	0.85748406	-1.31265310
Au	-1.06880508	-1.47201911	2.67873320
Au	3.26846125	-0.05480600	0.00000000
Au	-1.06880508	-1.47201911	-2.67873320
Au	-1.36288810	-1.71462513	-0.00000000

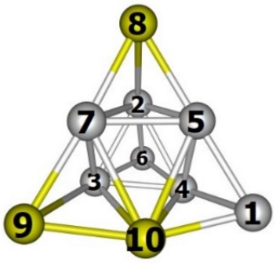

$Au_3Pt_7^{2+}$.A
(C_1)
septet

Pt	1.64118924	-1.49645925	-2.20534308
Pt	-1.81442278	-0.56948653	0.68856613
Pt	0.45863957	0.30807548	1.83011522
Pt	0.82764889	-1.71002417	0.17787910
Pt	-0.60946049	-0.42474314	-1.65762304
Pt	-0.49050561	-2.01063842	2.31353526
Pt	-0.65384360	1.72928908	-0.20001093
Au	-2.92398788	0.90690707	-1.25724001
Au	1.52805878	2.63519622	1.00440916
Au	2.02857306	0.57904839	-0.68229897

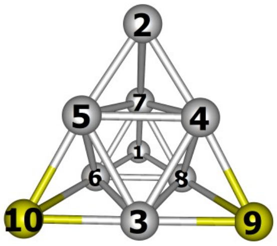

$Au_2Pt_8^{2+}$.A
(C_{2v})
nonet

Pt	0.00000000	2.55494218	1.82919213
Pt	-0.00000000	-2.55494218	1.82919213
Pt	-0.00000000	-0.00000000	-1.89513314
Pt	1.34354210	-1.39648710	0.03267100
Pt	-1.34354210	-1.39648710	0.03267100
Pt	-1.34354210	1.39648710	0.03267100
Pt	0.00000000	0.00000000	1.91763214
Pt	1.34354210	1.39648710	0.03267100
Au	2.62910219	-0.00000000	-1.88166114
Au	-2.62910219	0.00000000	-1.88166114

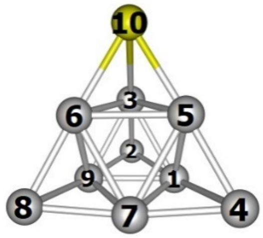

$AuPt_9^{2+}$.A
(C_{3v})
dectet

Pt	1.30834417	0.75537292	-1.18750708
Pt	-0.00000010	2.96050021	-1.01376208
Pt	-0.00000005	1.56040611	1.09768408
Pt	2.56386844	-1.48025002	-1.01376208
Pt	1.35135136	-0.78020301	1.09768408
Pt	-1.35135130	-0.78020310	1.09768408
Pt	0.00000005	-1.51074575	-1.18750708
Pt	-2.56386834	-1.48025020	-1.01376208
Pt	-1.30834422	0.75537283	-1.18750708
Au	0.00000000	0.00000000	3.26884623

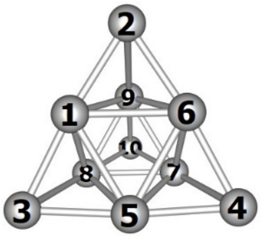

Pt_{10}^{2+}
(C_1)
11-et

Pt	0.00000000	0.00000000	-1.72233712
Pt	0.00000000	2.53834418	-1.89170114
Pt	-0.00000000	-2.53834418	-1.89170114
Pt	-2.59671018	0.00000000	1.71971012
Pt	-1.30402909	-1.48296211	0.06612600
Pt	-1.30402909	1.48296211	0.06612600
Pt	0.00000000	0.00000000	1.80181613
Pt	1.30402909	-1.48296211	0.06612600
Pt	1.30402909	1.48296211	0.06612600
Pt	2.59671018	-0.00000000	1.71971012
